# Wilforine attenuates inflammatory osteolysis by suppressing osteoclast fusion through JAK-STAT-stomatin immunoregulatory signaling

**DOI:** 10.3389/fimmu.2026.1789493

**Published:** 2026-03-13

**Authors:** Shaohui Geng, Yiwei Guan, Zi Ye, Dongdong Zhao, Yijin Jiang, Jingyuan Fu, Han Sheng, Shuhan Yang, Hongxu Liu, Fuwen Deng, Shasha Yu, Mureziya Yimingjiang, Yuanhao Wu, Chen Li, Guangrui Huang

**Affiliations:** 1 School of Life Science, Beijing University of Chinese Medicine, Beijing, China; 2 School of Chinese Materia Medica, Beijing University of Chinese Medicine, Beijing, China; 3 Dongzhimen Hospital, Beijing University of Chinese Medicine, Beijing, China; 4 School of Traditional Chinese Medicine, Beijing University of Chinese Medicine, Beijing, China; 5 School of Nursing, Beijing University of Chinese Medicine, Beijing, China; 6 First Teaching Hospital of Tianjin University of Traditional Chinese Medicine, Tianjin, China; 7 Shanghai GuangHua Hospital of Integrated Traditional Chinese and Western Medicine, Shanghai University of Traditional Chinese Medicine, Shanghai, China

**Keywords:** wilforine, bone erosion, immunometabolism, lipid rafts, osteoclast fusion, stomatin, SAPHO syndrome

## Abstract

**Background:**

Excessive osteoclast fusion and activation are central drivers of bone erosion in inflammatory osteopathies, which are closely linked to immune dysregulation. Wilforine, a natural compound, exhibits immunomodulatory and therapeutic potential, yet its precise mechanism of action—particularly its influence on the osteoclast membrane microenvironment and associated immune signaling—remains incompletely understood.

**Methods:**

We employed a multi-level strategy combining *in vivo* and *in vitro* functional validation with systematic multi-omics analysis. The *in vivo* efficacy of Wilforine was assessed in a Pstpip2^cmo^ mouse model via Micro−CT and histopathology. *In vitro* studies utilized osteoclast culture, TRAP staining, scanning electron microscopy, and immunofluorescence to evaluate cell fusion and protein localization. Preliminary target prediction was conducted using network pharmacology. The core mechanism was elucidated through integrated proteomics and transcriptomics, followed by targeted functional validation including β−cyclodextrin−mediated lipid raft disruption and immune−related pathway analysis.

**Results:**

Wilforine significantly alleviated bone erosion, marrow edema, and inflammatory infiltration *in vivo*, and potently inhibited osteoclast multinucleation *in vitro*. Multi-omics profiling revealed that Wilforine broadly reversed disease-associated dysregulation, specifically upregulating cholesterol metabolism and glycosphingolipid biosynthesis pathways while modulating key immune-inflammatory networks such as NF−κB. This systemic remodeling of the lipid metabolic landscape was functionally linked to the disruption of membrane lipid raft integrity—critical platforms for immune receptor signaling. Consequently, the raft-dependent localization and function of key fusion proteins (CD9, DC−STAMP) were impaired. Mechanistically, Wilforine exerted these effects by suppressing the JAK−STAT signaling pathway, a central regulator of immune and inflammatory responses, leading to the downregulation of the essential lipid raft scaffold protein Stomatin.

**Conclusion:**

This study defines a novel immunometabolic mechanism wherein Wilforine inhibits osteoclast fusion and bone resorption by reprogramming cellular lipid metabolism and disrupting the “JAK−STAT – Stomatin – Lipid Raft” functional axis. It highlights lipid rafts as a viable immunomodulatory microenvironment in bone disorders and provides a strong multi−omics−supported rationale for developing Wilforine as a bone−targeted immunotherapeutic agent.

## 1 Introduction

Bone marrow edema and bone erosion represent key pathological features of various inflammatory osteoarticular diseases, such as rheumatoid arthritis, SAPHO syndrome, ankylosing spondylitis, and inflammatory bowel diseases such as ulcerative colitis (1, 2). These changes are closely linked to the abnormal activation, excessive differentiation, and fusion of osteoclasts (3). As the primary bone-resorbing cells in the bone remodeling process, osteoclasts—under sustained stimulation by the inflammatory immune microenvironment—lead to bone microstructural destruction, bone marrow edema, and joint dysfunction, severely impacting patients’ quality of life (2, 4, 5). Current clinical medications primarily include bisphosphonates (e.g., zoledronic acid) (6, 7), biologics (e.g., anti-TNF-α monoclonal antibodies) (8–11), and small-molecule targeted drugs (e.g., JAK inhibitors) (12, 13). Although these agents can, to some extent, inhibit bone resorption or modulate immune-inflammatory responses, they remain associated with issues such as significant variability in efficacy, long-term side effects (e.g., osteonecrosis of the jaw, increased risk of infection), and high costs (14, 15). Therefore, exploring novel therapeutic strategies with innovative immunomodulatory mechanisms, good safety profiles, efficacy, and bone-targeting potential has become an important research direction in the field of bone metabolism diseases.

Wilforine, an active sesquiterpene alkaloid derived from the traditional Chinese medicine Tripterygium wilfordii, has been reported in previous studies to exhibit multiple pharmacological activities, including marked anti-inflammatory and immunomodulatory properties, as well as anti-tumor effects (16–20). It has demonstrated promising therapeutic potential, particularly in models of immune-mediated diseases such as rheumatoid arthritis and systemic lupus erythematosus (19, 21). However, its mechanism of action in osteoclast-driven bone loss under conditions of immune dysregulation remains to be systematically elucidated. In recent years, the role of cell membrane lipid rafts as key microdomains for signal transduction has become increasingly prominent in the fields of immunology and osteoimmunology, where they coordinate immune cell activation, inflammatory signaling, and intercellular communication (22–27). Lipid rafts are dynamic microdomains on the cell membrane enriched in cholesterol, sphingolipids, and specific proteins (22). They serve as assembly platforms for various signaling molecules (e.g., receptors, kinases, adaptor proteins) and are involved in regulating immune-relevant processes such as cell polarization, fusion, migration, and phagocytosis (28). In osteoclasts, lipid rafts not only promote differentiation by clustering receptors such as RANK and the immune receptor TREM2 but may also directly influence the formation of multinucleated osteoclasts and their bone-resorbing activity by organizing the spatial distribution of fusion-related proteins (e.g., DC-STAMP, CD9) (29–35). For example, studies indicate that key lipid raft proteins STOMATIN and FLOTILLIN-1 can co-localize with fusion molecules, and their expression levels positively correlate with the extent of osteoclast fusion (36–40). Furthermore, disruption of lipid raft structure using β-cyclodextrin significantly inhibits osteoclast fusion (30, 41, 42). Collectively, these findings suggest that targeting the assembly and function of lipid rafts—a nexus for immune and metabolic signaling— may represent a novel approach to regulating abnormal osteoclast activation and alleviating bone erosion.

PSTPIP2 (Proline-serine-threonine-phosphatase-interacting protein 2) is an adaptor protein associated with the cytoskeleton that is predominantly expressed in innate immune cells, such as macrophages and neutrophils, and serves a pivotal function in innate immune responses and inflammatory regulation (43). A growing body of evidence suggests that loss of function or reduced expression of PSTPIP2 is closely associated with the pathogenesis of various immune-mediated inflammatory diseases. For instance, in patients with rheumatoid arthritis (RA), the expression level of PSTPIP2 in synovial macrophages is negatively correlated with disease activity. PSTPIP2 regulates macrophage polarization and dynamics via estrogen receptor β (ERβ), helping to form a protective immunological barrier within the joint cavity and thereby resisting bone erosion (44). In experimental arthritis models, overexpression of PSTPIP2 significantly alleviates joint inflammation, reduces osteoclast numbers, and inhibits bone destruction (45). Furthermore, PSTPIP2 exhibits potent immunomodulatory and anti-inflammatory effects in models of organ inflammatory injury (e.g., liver injury, kidney injury, sepsis), through mechanisms including the suppression of key immune-inflammatory pathways like IL-1β and NF-κB, and modulation of macrophage polarization (46–48).

Of particular importance, mice carrying a specific mutation (Pstpip2cmo) spontaneously develop chronic multifocal osteomyelitis (CMO), whose clinical and pathological features highly resemble human SAPHO syndrome (Synovitis, Acne, Pustulosis, Hyperostosis, Osteitis) (49). Pstpip2cmo mice exhibit sterile bone inflammation primarily driven by the pro-inflammatory cytokine IL-1β, characterized by infiltration of neutrophils and monocytes/macrophages. This immune cell-driven inflammatory milieu is accompanied by significant osteoclast activation, enhanced bone resorption, and bone marrow edema, and ultimately leads to severe bone destruction and joint deformity. This model perfectly recapitulates the core pathological link of the “immune dysregulation–bone destruction” axis seen in SAPHO syndrome and similar inflammatory bone diseases. Therefore, the Pstpip2^cmo^ mouse model provides a genetically defined and immunologically relevant system to directly evaluate drug interventions against inflammatory bone destruction and to mechanistically explore drug actions on osteoclasts within their pathological immune-bone microenvironment.

**Figure 1:**
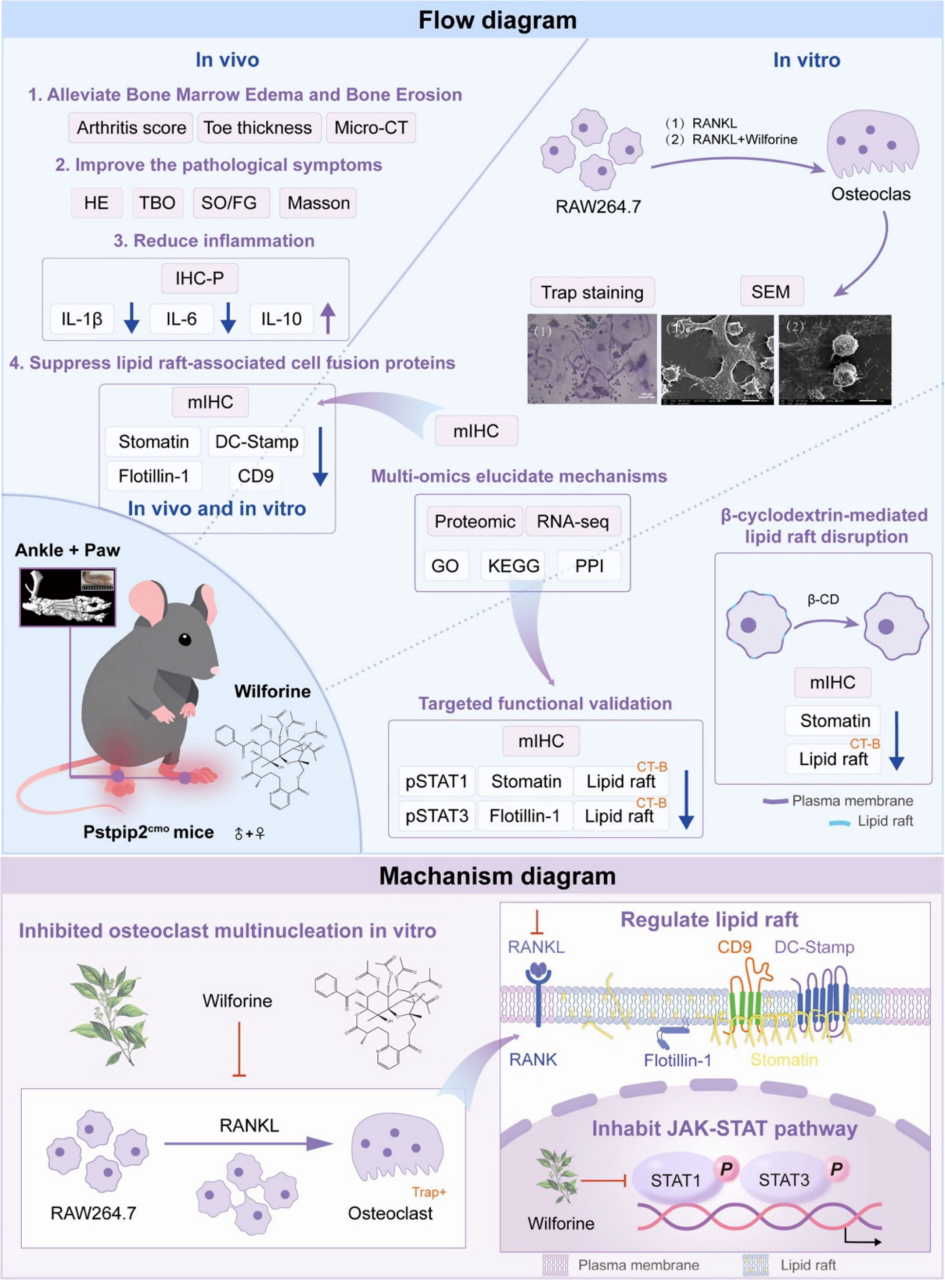
Schematic overview of the experimental workflow and the proposed mechanism by which Wilforine targets the immunomodulatory JAK-STAT-Stomatin-Lipid Raft axis to inhibit osteoclast fusion.

Based on this, the present study aims to investigate whether Wilforine alleviates bone marrow edema and bone erosion in the context of an inflammatory immune microenvironment by specifically targeting the structure and function of membrane lipid rafts—key platforms for immune signaling, thereby inhibiting osteoclast fusion and activation. As shown in **Figure 1**, by constructing a Pstpip2^cmo^ mouse model (simulating immune-mediated bone disorders) and employing a multi-level research approach integrating *in vivo* efficacy evaluation, high-resolution microscopic imaging, proteomics, transcriptomics, and gene/protein functional validation, this study systematically elucidates the bone-protective effects of Wilforine and its molecular mechanism of regulating osteoclast function via the “lipid raft–JAK-STAT–STOMATIN” signaling axis, a pathway critically involved in immune-metabolic crosstalk. This research not only provides new evidence for a deeper understanding of the role of lipid rafts in immunometabolic regulation of bone diseases but also offers an important experimental basis and theoretical support for the clinical application of Wilforine and the development of lipid raft-targeted immunomodulatory agents.

## 2 Materials and methods

### 2.1 Reagents and key materials

Wilforine was purchased from AbMole BioScience (USA, Cat# M6228−1g). β−Cyclodextrin was obtained from MedChemExpress. Lipid rafts were labeled using the Vybrant™ Alexa Fluor™ 555 Lipid Raft Labeling Kit (Thermo Fisher Scientific, V34404). RANKL was sourced from ABclonal Systems. The TRAP staining kit was purchased from Sigma−Aldrich (387A). Information on the primary antibodies used is provided in the corresponding methodology sections. All other general reagents were commercially sourced and used according to the manufacturers’ instructions.

### 2.2 Experimental animals and grouping

#### 2.2.1 Animals and grouping

The Pstpip2 gene knockout mice (C57BL/6N background, conventional knockout) were used as the disease model in this study and were supplied by Cyagen Biosciences (China). C57BL/6 mice were used as the normal controls. All mice were housed in the Laboratory Animal Center of Beijing University of Chinese Medicine under specific pathogen−free (SPF) conditions, with a 12−hour light/dark cycle and free access to food and water.

At the start of the experiment, both male and female mice were used at a 1:1 ratio, aged 6 weeks, with body weights of (25 ± 5) g. After a 7−day acclimation period, the animals were stratified by body weight to ensure baseline comparability and then randomly assigned to the following four groups using a random number generator: (1) Normal group (C57, n=4); (2) Model group (Pstpip2^cmo^, n=4); (3) Low−dose Wilforine group (Pstpip2^cmo^ + Wilforine 10 mg/kg, n=3); (4) High−dose Wilforine group (Pstpip2^cmo^ + Wilforine 20 mg/kg, n=4). Combined with the difficulty of obtaining animal models, the sample size is in line with the experimental design of animals used in the previously reported rare disease models (50, 51). All animal experiments were conducted in strict accordance with ethical guidelines for laboratory animals and were approved by the Animal Ethics Committee of Beijing University of Chinese Medicine (Approval No. BUCM−2024030105−1207).

#### 2.2.2 Drug administration

Wilforine was administered orally at doses of 10 mg/kg (low-dose) and 20 mg/kg (high-dose), respectively. The dosing volume was 10 mL/kg (approximately 300 μL per mouse based on an average body weight of 30 g). Administration was performed once daily for 28 consecutive days. Wilforine was dissolved in a vehicle consisting of 2% DMSO, 30% PEG300, 2% Tween 80, and 66% ddH_2_O. The working solution concentrations were 1 mg/mL for the low−dose group and 2 mg/mL for the high−dose group. The normal and model groups received an equal volume of the vehicle, following the same administration route, volume, and frequency as the treatment groups.

### 2.3 Paw inflammation scoring and paw thickness measurement

Referring to the arthritis scoring system and taking into account the main characteristics of plantar destruction, mice with the following four features will receive one point each: stiffness, fluid accumulation, toe thickness > 3.5 millimeters, and contracture. Scores were cumulative based on the number of features present. Throughout the experiment, the left and right paws and ankle joints of mice were scored dynamically on a scale of 0–4 points. Paw thickness was measured using a vernier caliper. Arthritis scores for mice in each group were evaluated every 3 days, and limb diameter was measured at the same intervals.

### 2.4 Micro-CT scanning and bone structure analysis

After 28 days of drug administration, the animals were euthanized under anesthesia. The ankle and paw joints, after removal of the skin, were promptly fixed in 4% paraformaldehyde solution. Micro−CT scanning of the joints and toes of Pstpip2cmo mice was performed using a SkyScan 1276 system (Bruker, Kontich, Belgium). The raw data were reconstructed three−dimensionally using NRecon software (Bruker), spatially corrected with DataViewer software (Bruker), and subsequently visualized in three dimensions using CTVox software (Bruker). To minimize bias, Micro-CT scanning and 3D reconstruction analysis were performed by investigators who were blinded to the group allocation.

Based on anatomical landmarks, representative regions of interest (ROIs) covering the joint and toe bone areas were selected. Qualitative observation and inter−group comparison were conducted, focusing on joint destruction, bone erosion, and abnormal bone structure.

### 2.5 Histological staining

Following fixation, the ankle and paw tissues were subjected to routine dehydration, embedded in paraffin, and sectioned to a thickness of approximately 4 μm. After deparaffinization and rehydration, the paraffin sections were subjected to Hematoxylin and Eosin (H&E) staining, Masson’s trichrome staining, Safranin O/Fast Green staining, and Toluidine Blue staining to evaluate morphological changes, collagen deposition, cartilage structure, and matrix composition.

#### 2.5.1 H&E staining

H&E staining was performed using a commercial kit (Servicebio, G1076). After staining with hematoxylin, differentiation, bluing, and counterstaining with eosin, sections were dehydrated, cleared, and mounted with neutral resin for observation of overall tissue architecture and inflammatory cell infiltration.

#### 2.5.2 Masson’s trichrome staining

Masson’s trichrome staining was carried out using a Masson staining kit (Servicebio, G1006). Sections were processed for nuclear staining, counterstaining, and differentiation, followed by dehydration, clearing, and mounting. This staining was used to assess collagen fiber deposition in and around the joints, with collagen fibers appearing blue and muscle fibers/cytoplasm appearing red.

#### 2.5.3 Safranin O/fast green staining

Safranin O/Fast Green staining was conducted using a bone tissue staining kit (Servicebio, G1053). Sections were sequentially stained with Fast Green and Safranin O, then rapidly dehydrated, cleared, and mounted to evaluate cartilage and bone structure. Cartilage matrix stains red or orange-red, while osteoid areas appear green.

#### 2.5.4 Toluidine blue staining

Toluidine Blue staining was performed using Toluidine Blue solution (Servicebio, G1032). After staining, sections were appropriately differentiated, dehydrated, and mounted to visualize cartilage matrix and extracellular components, with cartilage tissue staining purplish-blue.

All stained sections were observed under a bright-field microscope, and images were captured for qualitative histological comparison and analysis.

### 2.6 Immunohistochemistry and multiplex immunohistochemistry staining

After euthanasia, the ankle and paw tissues were harvested and fixed in neutral buffered formalin, followed by routine dehydration, paraffin embedding, and sectioning at a thickness of approximately 4 μm. Following deparaffinization and rehydration, paraffin sections were used for immunohistochemical and immunofluorescence staining.

#### 2.6.1 Immunohistochemical staining

Antigen retrieval was performed using Tris-EDTA buffer (pH 8.0) heated in a retrieval device (approximately 70 °C). After natural cooling, sections were washed with PBS (pH 7.4). Endogenous peroxidase activity was blocked by incubating sections with 3% hydrogen peroxide in the dark at room temperature, followed by PBS washes.

Sections were blocked with 3% BSA at room temperature for 30 min, then incubated with the following primary antibodies overnight at 4°C: IL-6 (1:800, GB11117), IL-1β (1:800, GB11113), and IL-10 (1:100, GB11108). After washing with PBS, HRP-conjugated secondary antibodies matching the species of the primary antibodies were applied and incubated at room temperature. Color development was performed using a DAB substrate kit, with reaction time monitored under a microscope and stopped by rinsing with running water. Sections were counterstained with hematoxylin, dehydrated through an ethanol gradient, cleared, and mounted. Immunohistochemical results were observed under an optical microscope; DAB-positive signals appeared brownish-yellow, and nuclei were counterstained blue. This method was used to evaluate the tissue expression and distribution of relevant inflammatory factors and proteins in joint and paw tissues.

#### 2.6.2 Multiplex immunohistochemical staining

Paraffin-embedded tissue sections were routinely deparaffinized and rehydrated, followed by permeabilization with 0.1% Triton X-100. Endogenous peroxidase activity was blocked by incubation with 3% H_2_O_2_ at room temperature in the dark. Antigen retrieval was then performed using sodium citrate buffer (1×) by heating at 95–100°C for 15–20 min, followed by natural cooling and washing with PBS. After blocking with 10% normal goat serum at 37°C for 30 min, multiplex immunofluorescence staining was performed using a sequential staining strategy on different sections according to the experimental design. For each section, the first primary antibody was incubated at 37°C for 1 h, followed immediately by incubation with the corresponding species-matched HRP-conjugated secondary antibody and signal amplification using the TSA system with the appropriate fluorophore. After completion of the first round of staining, sections were subjected to microwave treatment to remove antibody complexes and subsequently incubated overnight at 4°C with the second primary antibody, followed by incubation with the corresponding secondary antibody and TSA-based fluorescent signal development. Stomatin (1:200, ab166623) and Flotillin-1 (1:200, ab133497) were sequentially stained on the same section, while CD9 (1:1000, ab263019) and DC-STAMP (1:200, MABF39-I) were sequentially stained on another section. Nuclei were counterstained with DAPI, and sections were mounted with an anti-fade mounting medium. Fluorescent images were acquired using a fluorescence or confocal microscope to evaluate the tissue distribution and co-expression patterns of the target proteins in ankle joint and paw tissues.

### 2.7 *In vitro* induction and treatment of osteoclasts

Osteoclast precursor cells were RAW264.7, a murine macrophage cell line. Cells were cultured in DMEM medium supplemented with approximately 9% (v/v) fetal bovine serum under 37°C with 5% CO_2_. Osteoclast differentiation was induced by RANKL (R&D Systems) at a final concentration of 50 ng/mL for 6 days. The culture medium was refreshed every 2 days with replenishment of RANKL.

To interfere with lipid raft structure, cells were treated with β-CD (1 mM, 12 h; MedChemExpress) or Wilforine (1 mM, 12 h; AbMole BioScience), using 0.1% DMSO as the vehicle control.

### 2.8 TRAP staining

Osteoclasts were identified by tartrate-resistant acid phosphatase (TRAP) staining using a commercial kit (Solarbio, TRAP staining kit, Cat# G1492) following the manufacturer’s instructions. After fixation, cells were incubated with TRAP reaction solution at 37°C for 45–60 min. After staining, samples were rinsed with distilled water and, if required, counterstained with hematoxylin. Staining results were observed under an optical microscope, with TRAP-positive cells appearing purplish-red.

### 2.9 Lipid raft labeling and immunofluorescence colocalization

Lipid rafts were labeled using the Vybrant Lipid Raft Labeling Kit. Adding 100 μM Wilforine intervention when cells show a tendency to differentiate. After osteoclast differentiation is completed, carry out subsequent experimental steps. Cells were blocked with 5% BSA (or normal goat serum) in the dark at room temperature for 30–60 min. The following primary antibodies were applied and incubated in the dark at room temperature for 1 h: Stomatin (1:200, ab166623), Flotillin-1 (1:200, ab133497), CD9 (1:1000, ab263019), and DC-STAMP (1:200, MABF39-I). Unbound primary antibodies were removed by washing with PBS.

Fluorescent secondary antibodies—goat anti-mouse IgG H&L (Alexa Fluor^®^ 488, ab150117, 1:1000) or was then applied and incubated in the dark at room temperature for 1 h. After washing with PBS to remove unbound secondary antibodies, nuclei were stained with DAPI mounting medium for 5–10 min in the dark. Samples were observed under a fluorescence microscope, and images were captured.

### 2.10 Immunofluorescence staining of cells

Cells were seeded onto confocal culture dishes, adding 100 μM Wilforine intervention when cells show a tendency to differentiate. After osteoclast differentiation is completed, carry out subsequent experimental steps. Cells were fixed with 4% paraformaldehyde at room temperature for 15 min. After washing with PBS, cells were permeabilized with Triton X-100 and blocked with blocking buffer at room temperature.

Cells were then incubated with primary antibodies, including recombinant anti-STAT3 (phospho-Y705) antibody (Abcam, ab76315) and recombinant anti-STAT1 (phospho-S727) antibody (Abcam, ab109461), for 60 min at room temperature or overnight at 4°C. After PBS washes, cells were incubated with the corresponding fluorescently labeled secondary antibodies at room temperature in the dark. Nuclei were counterstained with DAPI, and cells were mounted with an anti-fade mounting medium. Immunofluorescence images were acquired using a confocal fluorescence microscope.

### 2.11 Network pharmacology methodology

#### 2.11.1 Identification of active compounds

This study selected Wilforine, a major alkaloid component of Tripterygium wilfordii, as the research subject. The two−dimensional chemical structure of Wilforine was obtained from the PubChem database (https://pubchem.ncbi.nlm.nih.gov) and subsequently used for target prediction and molecular docking analysis.

#### 2.11.2 Prediction of potential targets of wilforine

Potential targets of Wilforine were predicted using multiple publicly available small−molecule target prediction platforms, including SwissTargetPrediction (https://www.swisstargetprediction.ch/) and PharmMapper (http://www.lilab-ecust.cn/pharmmapper/). The target results predicted by each platform were integrated and imported into the UniProt database (https://www.uniprot.org) for gene−name standardization, with the species restricted to Homo sapiens. Duplicate targets were removed to generate the final set of Wilforine−related targets.

#### 2.11.3 Collection of disease−related targets

Disease−related targets were retrieved from the following disease−gene databases: GeneCards (https://www.genecards.org/) and DisGeNET (https://www.disgenet.org/). Searches were performed using relevant disease keywords, and targets with higher relevance scores were selected. The obtained targets were consolidated, standardized, and deduplicated to construct a disease−related target database.

#### 2.11.4 Screening of overlapping targets

Wilforine−related targets were compared with disease−related targets. The Venny 2.1 online tool (https://bioinfogp.cnb.csic.es/tools/venny/) was used to generate a Venn diagram and identify the overlapping targets, which served as potential therapeutic targets for subsequent analysis.

#### 2.11.5 Construction of the protein–protein interaction network

The overlapping targets were imported into the STRING database (https://string-db.org/) to construct a protein–protein interaction network, with the species limited to Homo sapiens and the minimum interaction confidence set to the default value. After downloading the PPI network results, Cytoscape 3.9.1 software (https://cytoscape.org/) was used for network visualization and topological analysis to identify key targets.

#### 2.11.6 GO functional and KEGG pathway enrichment analysis

The Metascape online analysis platform (https://metascape.org) was employed to perform Gene Ontology (GO) functional enrichment analysis (BP, CC, MF) and KEGG pathway enrichment analysis on the overlapping targets. Statistically significant enrichment results (P < 0.05) were selected and visualized.

#### 2.11.7 Molecular docking analysis

Key targets were selected based on the results of network and enrichment analyses. The three−dimensional structures of the target proteins were obtained from the Protein Data Bank (PDB) database (https://www.rcsb.org/). Molecular docking analysis between Wilforine and the core targets was performed using the DockEasy molecular docking platform (https://www.dockeasy.cn/) to evaluate binding energy and interaction patterns.

### 2.12 Transcriptomic analysis

Transcriptomic analysis was performed on synovial tissue from the mouse ankle joints. Total RNA was extracted from these tissues and subjected to RNA sequencing (RNA-seq) to assess the gene expression profiles. Based on quantitative expression data, differentially expressed genes (DEGs) between groups were identified using DESeq2 software, with thresholds set at |log_2_FC| ≥ 1 and *P−value* < 0.05, to investigate the mechanism by which Wilforine inhibits osteoclast differentiation.

### 2.13 Proteomic analysis

Proteomic analysis was conducted on samples from each experimental group. Following total protein extraction, liquid chromatography–tandem mass spectrometry (LC–MS/MS) was employed to detect and quantify the protein expression profiles. Based on the quantitative protein data, differentially expressed proteins between groups were screened using statistical analysis, with thresholds set at |log_2_ fold change| ≥ 1 and *P−value* < 0.05. This proteomic analysis was performed to evaluate changes in the protein expression profiles after Wilforine treatment, providing a basis for subsequent mechanistic studies.

### 2.14 Statistical analysis

All data are presented as mean ± standard deviation (SD). Comparisons between two groups were performed using the Student’s t-test. Comparisons among multiple groups were conducted using one-way analysis of variance (ANOVA), followed by Dunnett’s test for *post hoc* analysis. All statistical analyses were carried out using Prism software version 9.5.0 (GraphPad Software Inc., San Diego, CA, USA). A *P-value* < 0.05 was considered statistically significant.

## 3 Results

### 3.1 Alleviating effects of wilforine on bone marrow edema and bone erosion in Pstpip2cmo mice

**Figure 2:**
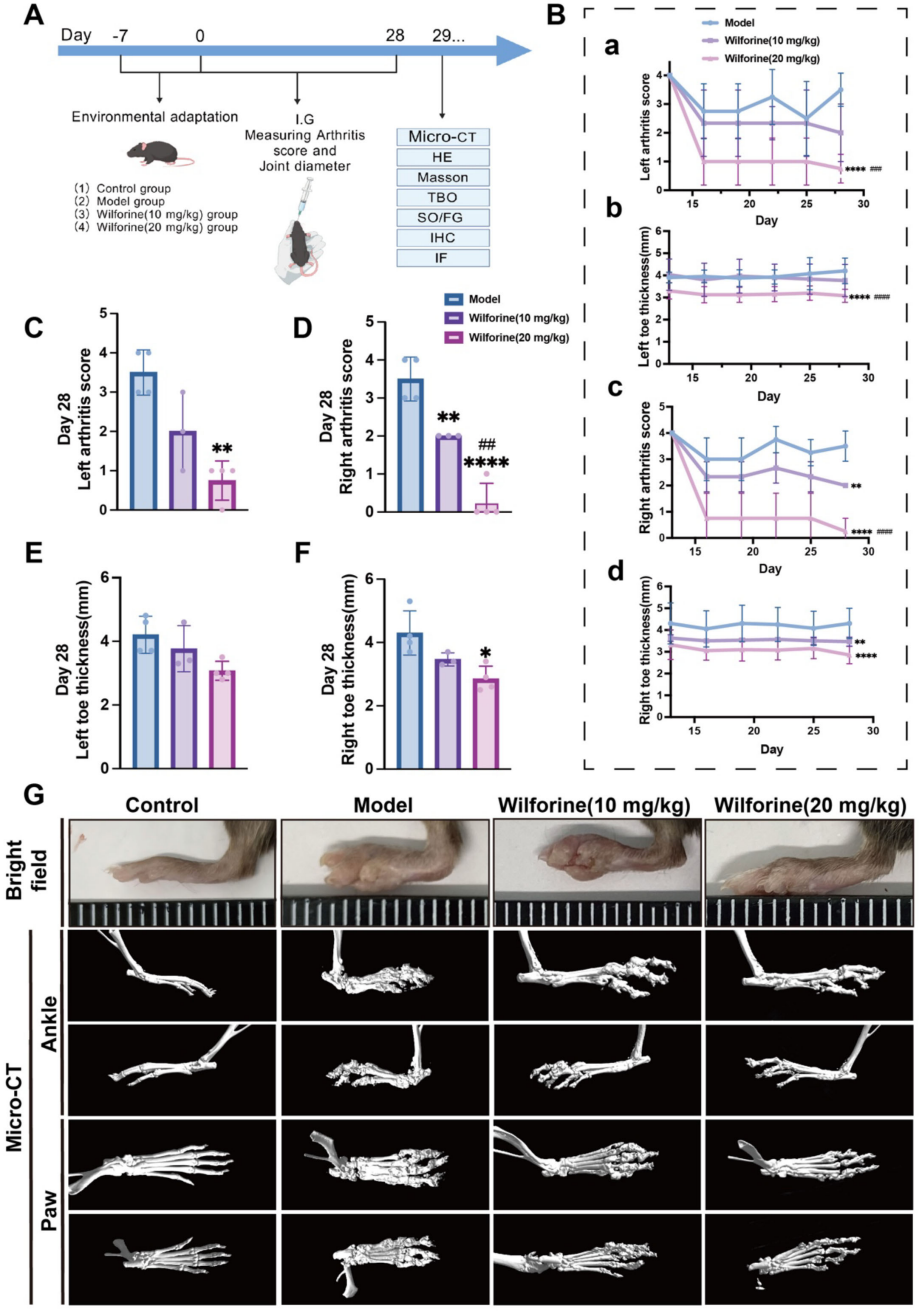
Analysis of the therapeutic effect of Wilforine on arthritis in Pstpip2^cmo^ mice. **(A)** Schematic diagram of the animal experimental protocol. **(B)** Dynamic changes in arthritis scores and left/right paw thickness of mice in each group. The panels show the changes in arthritis scores **(a, c)** and paw thickness **(b, d)** of the left and right paws in each group during the experiment. **(C–F)** Comparison of arthritis scores **(C, D)** and paw thickness **(E, F)** of the left and right paws measured in each group after the 28-day treatment. **(G)** Bright-field images and Micro-CT scans of mice from each group. ^#^Compared with low-dose group; ^*^Compared with model group. (mean ± SD, n = 3–4, ^###^*P* < 0.001, ^####^*P* < 0.0001, *P < 0.05, **P < 0.01, ****P < 0.0001).

Pstpip2^cmo^ mice exhibiting evident joint swelling received a 28-day oral administration of Wilforine, with therapeutic efficacy assessed via Micro-CT and histopathological analysis. As shown in **Figures 2A**, Wilforine treatment effectively reduced immune-mediated paw inflammation and swelling. Therapeutic efficacy was assessed using methods including the Micro-CT and histological staining. Analysis of arthritis scores and changes in paw thickness for the left and right toes/ankles of mice from each group revealed that Wilforine was effective in mitigating inflammation and reducing paw swelling. As shown in **Figures 2B**, compared to the model group, Wilforine treatment groups (particularly the high-dose group at 20 mg/kg) showed significant efficacy in lowering arthritis scores and reducing paw swelling, with the high-dose group demonstrating superior effects to the low-dose group (10 mg/kg). **Figures 2C–F** present comparisons of arthritis scores and paw thickness for the left and right paws of mice from each group after the 28-day treatment. Both arthritis scores and paw thickness were lower in the Wilforine-treated groups than in the model group, with the high-dose group showing better therapeutic outcomes across all metrics. Statistical analysis indicated significant differences in multiple indicators for the Wilforine treatment groups (*P* < 0.01, *P* < 0.0001). These results demonstrate that Wilforine significantly alleviated bone marrow edema and paw swelling in Pstpip2^cmo^ mice, with its efficacy showing a clear dose-dependent trend.

**Figure 2G** shows bright-field images and Micro-CT scans of mice from each group. In the bright-field images, pronounced paw swelling was observed in the model group, whereas swelling was markedly reduced in the Wilforine-treated groups. Micro-CT scans further confirmed this finding: mice in the model group exhibited severe inflammation-driven bone damage and joint fusion, while treated mice showed clearer joint margins and significantly reduced areas of bone destruction, suggesting a beneficial bone-repair effect of Wilforine.

### 3.2 Effect of wilforine on ankle and paw joint pathology in Pstpip2^cmo^ mice

**Figure 3:**
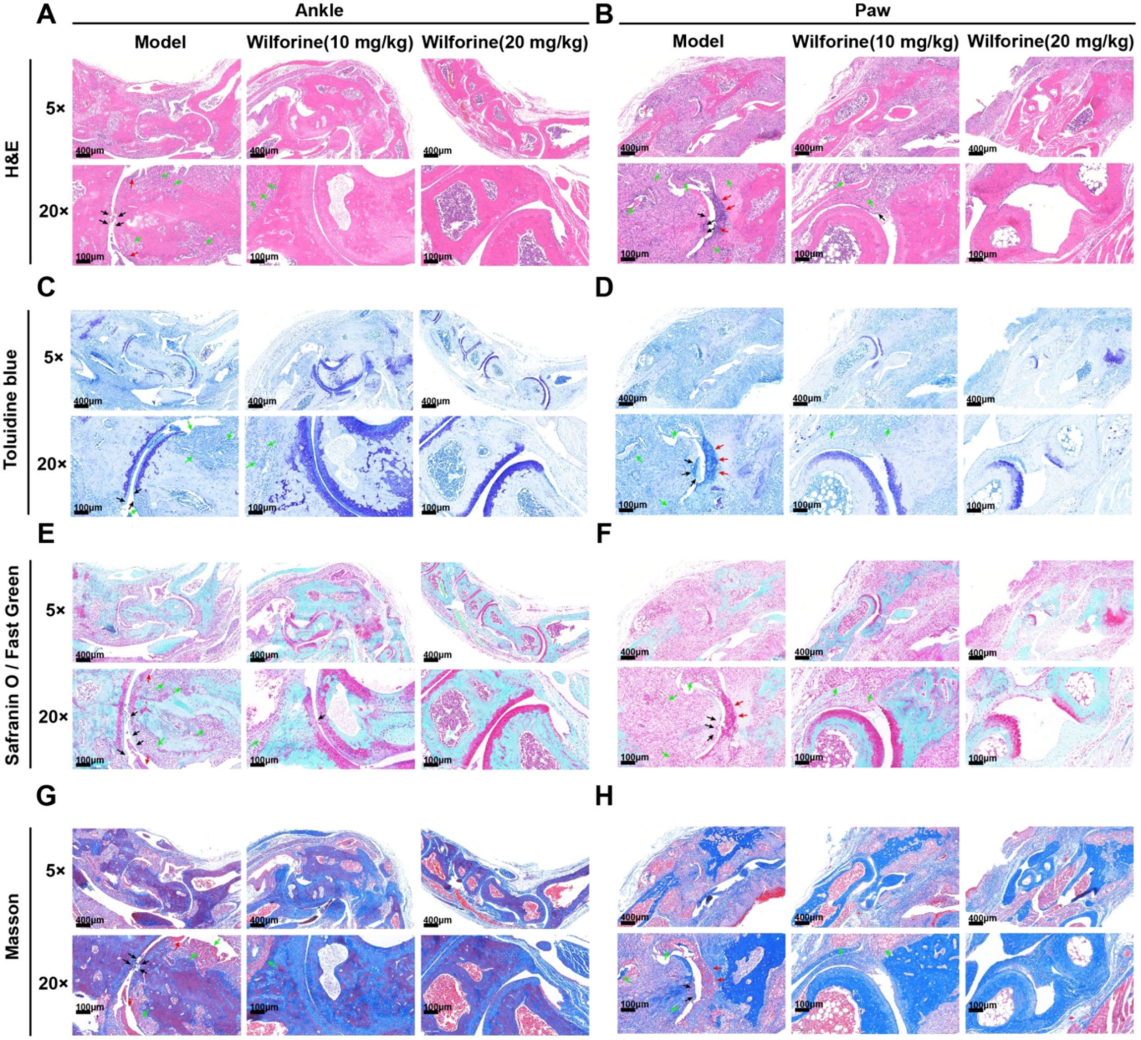
Histopathological staining of ankle and paw joints in Pstpip2cmo mice after treatment with wilforine **(A)** H&E staining of ankle joint tissues from the model group, low-dose Wilforine (10 mg/kg) group, and high-dose Wilforine (20 mg/kg) group; **(B)** H&E staining of paw joint tissues from the model group, low-dose Wilforine (10 mg/kg) group, and high-dose Wilforine (20 mg/kg) group; **(C)** Toluidine blue staining of ankle joint tissues from the model group, low-dose Wilforine (10 mg/kg) group, and high-dose Wilforine (20 mg/kg) group; **(D)** Toluidine blue staining of paw joint tissues from the model group, low-dose Wilforine (10 mg/kg) group, and high-dose Wilforine (20 mg/kg) group; **(E)** Safranin O/Fast Green staining of ankle joint tissues from the model group, low-dose Wilforine (10 mg/kg) group, and high-dose Wilforine (20 mg/kg) group; **(F)** Safranin O/Fast Green staining of paw joint tissues from the model group, low-dose Wilforine (10 mg/kg) group, and high-dose Wilforine (20 mg/kg) group; **(G)** Masson’s trichrome staining of ankle joint tissues from the model group, low-dose Wilforine (10 mg/kg) group, and high-dose Wilforine (20 mg/kg) group; **(H)** Masson’s trichrome staining of paw joint tissues from the model group, low-dose Wilforine (10 mg/kg) group, and high-dose Wilforine (20 mg/kg) group; (Black arrows indicate joint space damage; red arrows indicate synovial hyperplasia; green arrows indicate inflammatory cell infiltration.).

As shown in **Figures 3A, B**, HE staining revealed that in the model group, the surface layer of cartilage tissue in the ankle and paw joints showed obvious damage, with rough and uneven articular surfaces, disordered arrangement, and reduced number of chondrocytes, marked synovial hyperplasia and thickening, and extensive immune cell infiltration within the synovium and sub-synovial layers. Compared with the model group, the medium- and high-dose wilforine groups exhibited reduced erosion of the articular cartilage surface and less synovial hyperplasia, along with more regular chondrocyte arrangement. The high-dose group showed more pronounced improvement in joint tissue structure.

As shown in **Figures 3C, D**, safranin O–fast green staining indicated that in the model group, the superficial cartilage of both paw and ankle joints showed loss of safranin O staining, accompanied by a decrease in the number of chondrocytes. The paw joint cartilage surface was rough with the disappearance of the tidemark, while the ankle joint cartilage layer exhibited bone surface defects and an irregular tidemark. In the medium-dose group, the loss of safranin O staining in the cartilage layer of both paw and ankle joints was alleviated, and the number of chondrocytes increased compared with the model group. The paw joint cartilage surface was smooth but with an irregular tidemark, whereas the ankle joint cartilage surface remained rough with a burr-like appearance and an irregular tidemark. In the high-dose group, the cartilage layer of both paw and ankle joints exhibited uniform and intense safranin O staining, a normal number of chondrocytes, a smooth articular cartilage surface, and a clear tidemark.

As shown in **Figures 3E, F**, toluidine blue staining demonstrated that in the model group, proteoglycan content in the cartilage matrix was reduced, with visible erosion and fissure formation on the cartilage surface, indicating disruption of cartilage structural integrity. With increasing doses of wilforine, the depth of staining in the articular cartilage gradually recovered, erosion on the cartilage surface decreased and became smoother and more integrated, and chondrocyte arrangement became more regular and well-organized. These findings demonstrate that wilforine dose-dependently ameliorated cartilage matrix degradation and tissue morphological damage induced by arthritis.

As shown in **Figures 3G, H**, Masson staining revealed that in the model group, substantial blue-stained collagen fiber deposition was observed in the synovium and periarticular tissues, along with obvious fibrous tissue hyperplasia, disordered collagen fiber arrangement, and unclear tissue layers in the joint region. In the medium-dose group, periarticular blue-stained collagen deposition was reduced, while in the high-dose group, the tissue structure was clearer and the degree of periarticular fibrosis was further reduced. Taken together, these histopathological results demonstrate that Wilforine effectively attenuates immune-driven cartilage degradation, synovitis, and periarticular fibrosis in Pstpip2^cmo^ mice in a dose-dependent manner.

### 3.3 Effect of wilforine on the expression of inflammatory factors in the ankle and paw joints of Pstpip2^cmo^ mice

**Figure 4:**
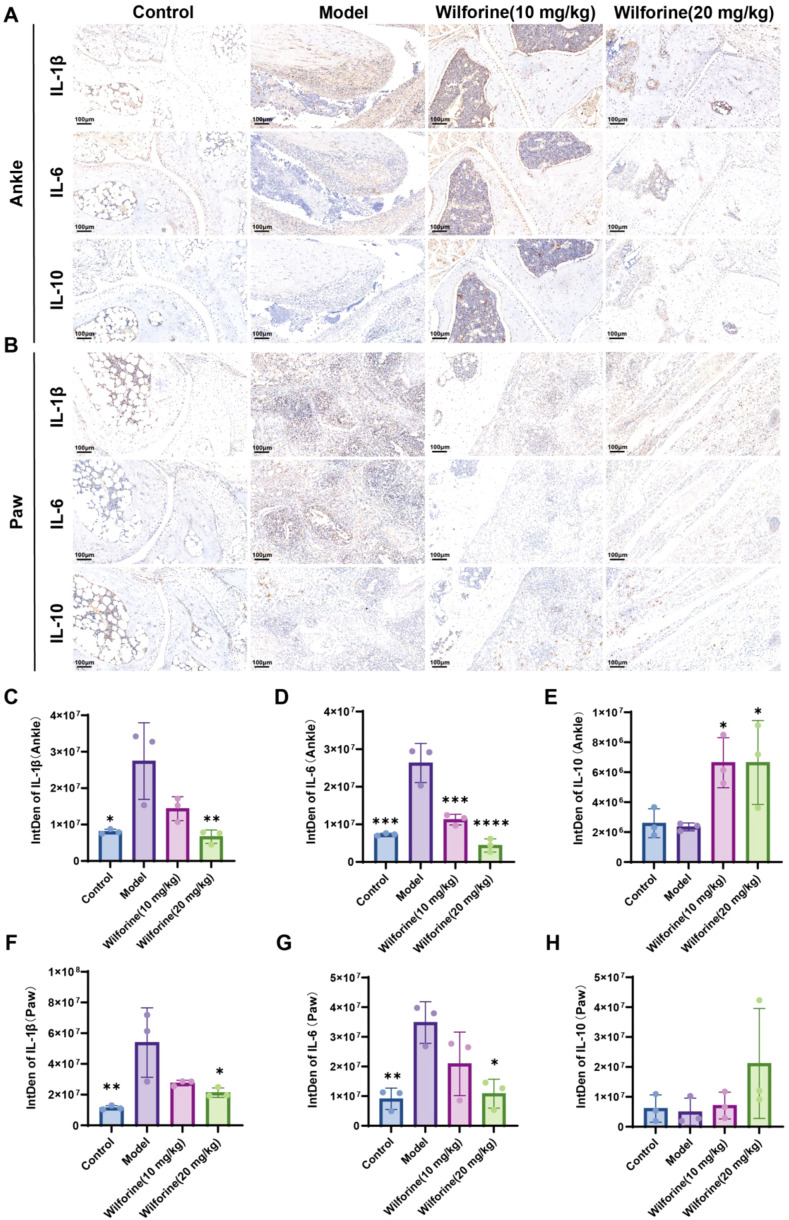
Wilforine suppresses the expression of inflammatory cytokines in ankle and paw tissues of Pstpip2cmo mice. **(A)** Representative IHC staining of IL-1β, IL-6, and IL-10 in ankle tissues from each group; **(B)** Representative IHC staining of IL-1β, IL-6, and IL-10 in paw (plantar) tissues from each group. Scale bar = 100 μm; **(C–E)** Quantification of IL-1β, IL-6, and IL-10 staining signals in ankle tissues (IntDen); **(F–H)** Quantification of IL-1β, IL-6, and IL-10 staining signals in paw tissues (IntDen). Data are presented as mean ± SD, n = 3. ^*^Compared with model group, ^*^P < 0.05, ^**^P < 0.01, ^***^P < 0.001, ^****^P < 0.0001.

To elucidate the immunomodulatory mechanism of Wilforine, we analyzed the expression of key cytokines at joint sites via immunohistochemistry. Assessment of pro-inflammatory immune mediators revealed that joints and bone marrow of Pstpip2^cmo^ mice exhibited substantial immune cell infiltration, accompanied by significantly elevated expression of IL−1β and IL−6 compared with the Control group (*P* < 0.05, *P* < 0.001; **Figures 4A, C, D**). Notably, high−dose Wilforine treatment significantly suppressed the expression of these pro−inflammatory cytokines (*P* < 0.01, *P* < 0.0001), indicating potent local anti−inflammatory activity within the osteoarticular immune microenvironment.

Similarly, in the plantar region, Pstpip2^cmo^ mice showed pronounced inflammatory infiltration and markedly increased levels of IL−1β and IL−6 (*P* < 0.01, *P* < 0.01; **Figures 4B, F, G**), which were effectively downregulated by high−dose Wilforine (*P* < 0.05, *P* < 0.05). Furthermore, Wilforine enhanced the expression of the anti−inflammatory cytokine IL−10 in joint tissues at higher doses (*P* < 0.05; **Figures 4A, B, E, H**).

Collectively, these results demonstrate that Wilforine restores immune balance in inflammatory joints by concurrently inhibiting pro−inflammatory cytokine production and promoting anti−inflammatory signaling, highlighting its role as a multi−target immunomodulator in the context of osteoimmunological pathology.

### 3.4 Wilforine regulate the lipid raft immunosignaling platform to suppress osteoclast fusion in inflammatory bone diseases

Excessive activation and fusion of osteoclasts represent a critical pathological link in inflammatory joint destruction, a process tightly regulated by immune signaling. Lipid rafts on the cell membrane are cholesterol- and sphingolipid-enriched microdomains that serve as crucial hubs for immune signal transduction and play a key role in immune cell communication and activation. Recent evidence highlights that lipid rafts and their associated scaffold proteins are pivotal in regulating the differentiation and function of bone cells within an inflammatory milieu.

**Figure 5:**
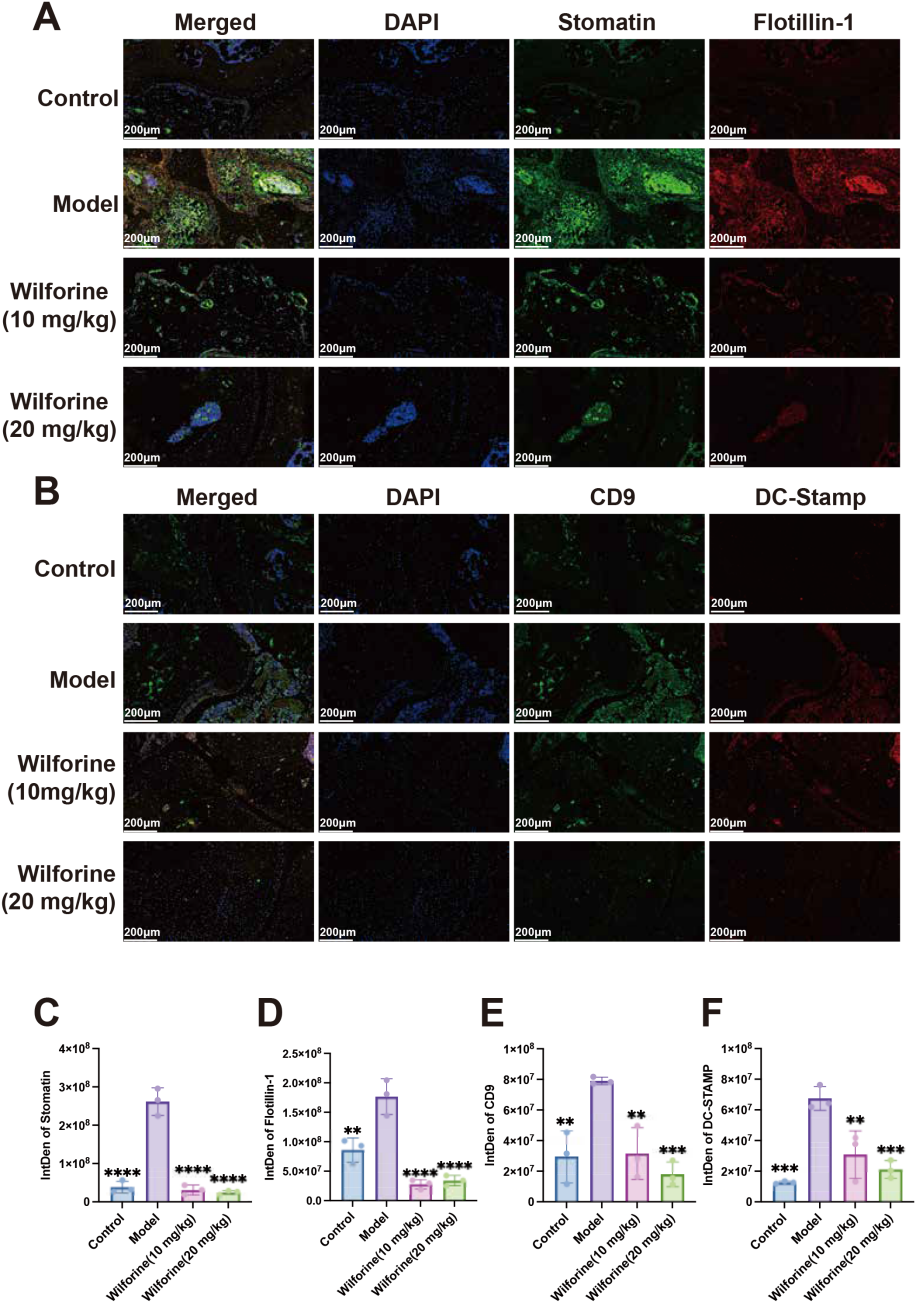
Wilforine reduces the expression of lipid raft–associated proteins and osteoclast fusion–related proteins in joint tissues. **(A)** mIHC images showing Stomatin and Flotillin-1 expression in joint tissues: Merged, DAPI, Stomatin, and Flotillin-1; **(B)** mIHC images showing CD9 and DC-STAMP expression in joint tissues: Merged, DAPI, CD9, and DC-STAMP. Scale bar = 200 μm; **(C–F)** Quantification of fluorescence intensity (IntDen) for Stomatin, Flotillin-1, CD9, and DC-STAMP. Data are presented as mean ± SD, n = 3. ^*^Compared with model group, ^**^P < 0.01, ^***^P < 0.001, ^****^P < 0.0001.

To investigate the mechanism by which Wilforine alleviates joint destruction in Pstpip2^cmo^ mice, we assessed the expression of key membrane proteins involved in osteoclast fusion and immune signaling in joint tissues using multiplex immunofluorescence. As shown in **Figures 5A, C, D**, the expression of lipid raft scaffold proteins Stomatin and Flotillin−1 was significantly increased in the joint tissues of the model group compared with the Control group (*P* < 0.0001, *P* < 0.01) consistent with enhanced membrane signaling activity under inflammatory conditions. Notably, high−dose Wilforine treatment significantly downregulated the expression of both Stomatin and Flotillin−1 compared with the model group (*P* < 0.0001, *P* < 0.0001).

As shown in **Figures 5B, E, F**, the expression of CD9 and DC−STAMP proteins was significantly elevated in the joint tissues of the model group compared with the Control group (*P* < 0.01, *P* < 0.001). However, after high−dose Wilforine treatment, the expression of Stomatin and Flotillin−1 proteins was significantly lower than that in the model group (*P* < 0.001, *P* < 0.001). Taken together, these findings demonstrate that Wilforine’s therapeutic effect involves the coordinated downregulation of both lipid raft organizers and critical fusogenic proteins, thereby disrupting the membrane-associated signaling platform necessary for excessive osteoclast fusion and subsequent inflammatory bone loss.

### 3.5 Wilforine inhibits osteoclast fusion *in vitro* by suppressing lipid raft-associated proteins

**Figure 6:**
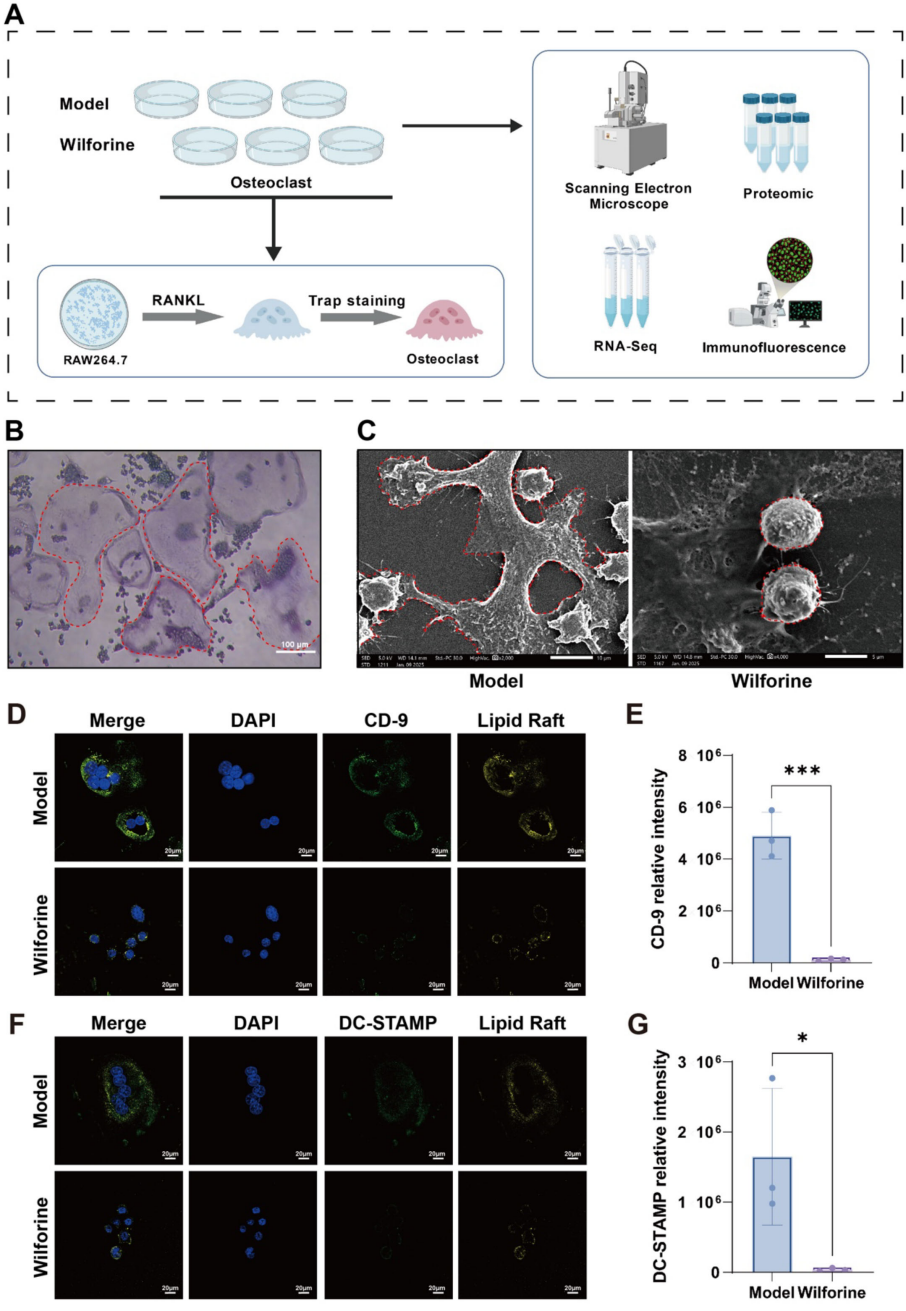
Wilforine inhibits osteoclast fusion by suppressing lipid raft-associated cell fusion proteins in RAW264.7 cells. **(A)** Schematic diagram of RAW264.7 cell differentiation into osteoclasts and subsequent experimental procedures; **(B)** Osteoclasts showing positive TRAP staining; **(C)** Scanning electron microscopy (SEM) images of osteoclast fusion with or without Wilforine treatment; **(D)** Suppression of CD9 protein colocalization with lipid raft markers by Wilforine in osteoclasts; **(E)** Quantitative analysis of CD9 protein expression inhibited by Wilforine in osteoclasts; **(F)** Suppression of DC-STAMP protein colocalization with lipid raft markers by Wilforine in osteoclasts; **(G)** Quantitative analysis of DC-STAMP protein expression inhibited by Wilforine in osteoclasts; (mean ± SD, n = 3, ^*^P < 0.05, ^***^P < 0.001).

To further elucidate the cellular mechanism through which Wilforine inhibits osteoclastogenesis, the murine macrophage cell line RAW264.7—a model for innate immune cells— was stimulated to differentiate into osteoclasts under pro-inflammatory conditions. As shown in **Figures 6A**, cells were divided into a Model group and a Wilforine-treated group, and TRAP (tartrate-resistant acid phosphatase) staining was used to confirm successful osteoclast differentiation. As shown in **Figures 6B**, TRAP-stained osteoclasts exhibited typical dark red or purplish-red coloration and multinucleation, indicating successful differentiation. To more directly observe the fusion characteristics of osteoclasts, high-resolution imaging analysis was performed using scanning electron microscopy (SEM). SEM results revealed that osteoclasts in the control group displayed typical multinuclear fusion features, with clearly visible intercellular membrane fusion areas. In contrast, the Wilforine-treated group showed significantly reduced osteoclast fusion and markedly fewer intercellular membrane fusion regions (**Figure 6C**). These results indicate that Wilforine can significantly inhibit the fusion process of osteoclasts, likely through its effects on the structure of cell membrane lipid rafts.

Furthermore, we assessed the colocalization relationship between cell fusion-related proteins and lipid rafts using immunofluorescence. As shown in **Figures 6D, E**, CD9 protein showed clear colocalization with the lipid raft marker, and Wilforine significantly reduced the expression of the cell fusion-related protein CD9 (*P* < 0.001). Similarly, DC-STAMP protein exhibited evident colocalization with the lipid raft marker, and Wilforine significantly decreased the expression of the cell fusion-related protein DC-STAMP (*P* < 0.05, **Figures 6F, G**). In summary, these findings demonstrate that Wilforine inhibits osteoclast fusion *in vitro*, an effect associated with the suppression of both lipid raft-associated proteins and cell fusion-related proteins.

In summary, these *in vitro* findings demonstrate that Wilforine directly inhibits the membrane fusion stage of osteoclastogenesis from immune-cell precursors. The mechanism involves the disruption of a functional immunoregulatory lipid raft microenvironment, leading to the coordinated downregulation and altered localization of critical fusion proteins (CD9 and DC-STAMP). This provides direct cellular evidence that Wilforine targets membrane-based signaling hubs to suppress the formation of multinucleated osteoclasts within an immune-inflammatory context.

### 3.6 Proteomic profiling demonstrates wilforine’s impact on membrane rafts and immune pathways

Building upon network pharmacology predictions, which suggested Wilforine’s multi-target potential in bone-destructive pathologies such as SAPHO syndrome, we employed an integrated proteomic and transcriptomic strategy to delineate its systemic mechanism. Initial in silico analyses identified core targets (e.g., EGFR, SRC, CTSK) associated with signal transduction, cell adhesion, inflammatory-immune responses, and osteoclast function. Molecular docking supported the potential binding of Wilforine to these targets(**Supplementary Figures 1-6**). To move beyond prediction and uncover the downstream immunometabolic landscape, we performed multi−omics profiling in a relevant disease model.

**Figure 7:**
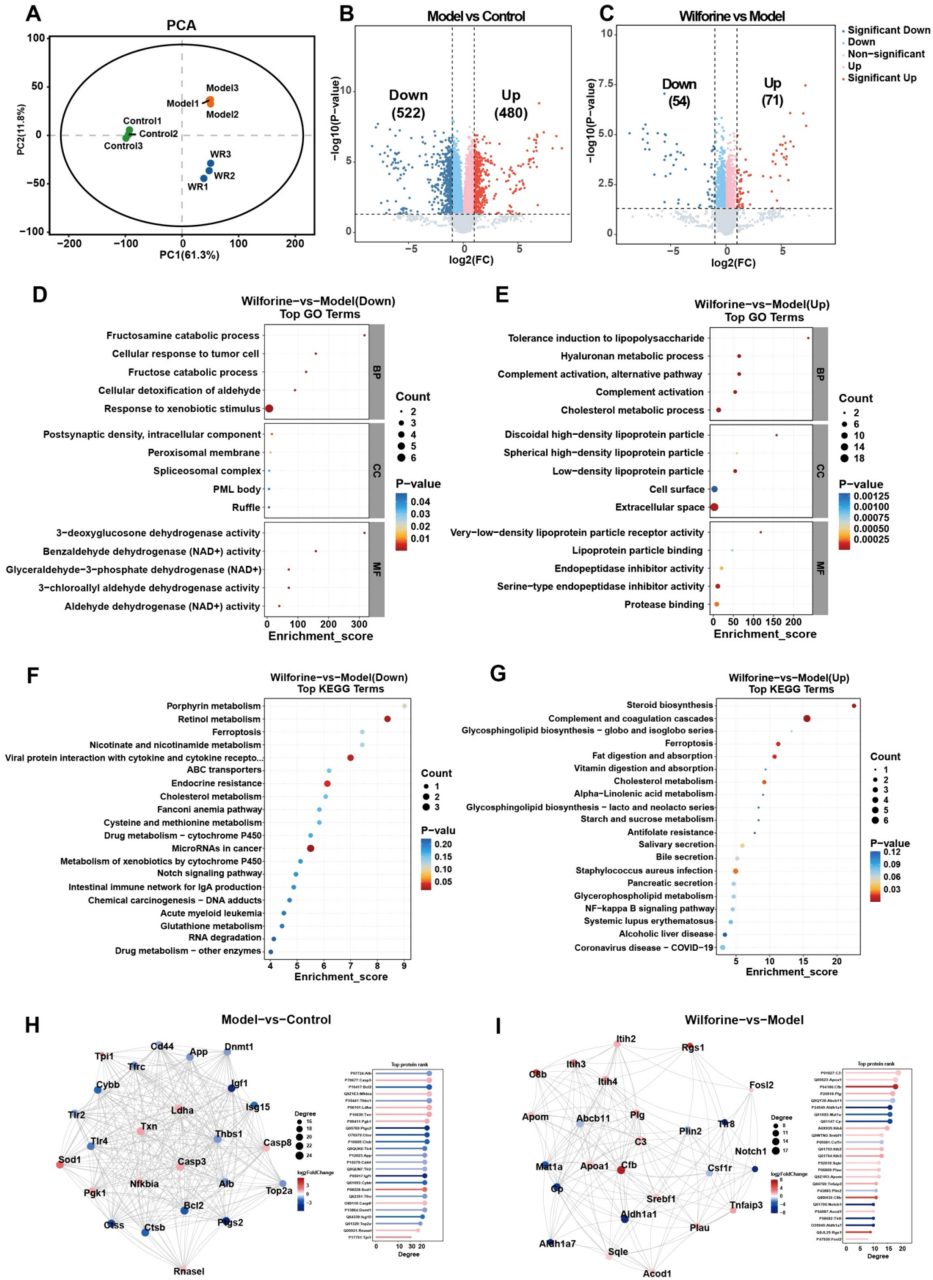
Quantitative proteomic analysis (Pro DIA) between the Wilforine-treated group and the model group. **(A)** Principal component analysis Control, Model, and Wilforine-treated groups; **(B)** Volcano plot of differentially expressed proteins between the Model and Control groups; **(C)** Volcano plot of differentially expressed proteins between the Wilforine-treated and Model groups; **(D, E)** GO functional enrichment analysis of differentially expressed proteins between the Wilforine-treated and Model groups; **(F, G)** KEGG pathway enrichment analysis of differentially expressed proteins between the Wilforine-treated and Model groups; **(H)** Protein-protein interaction (PPI) network of differentially expressed proteins between the Model and Control groups; **(I)** Protein-protein interaction (PPI) network of differentially expressed proteins between the Wilforine-treated and Model groups.

Proteomic analysis revealed that wilforine significantly reshaped the protein expression profile in osteoclasts from the SAPHO syndrome bone marrow edema model. PCA results showed that samples from the Control, Model, and Wilforine groups were clearly separated into three independent clusters in two-dimensional space (**Figure 7A**), confirming distinct proteomic characteristics. Differential protein expression analysis indicated that compared to the Control group, the Model group had 1,002 significantly altered proteins (522 downregulated and 480 upregulated) (**Figure 7B**). After wilforine treatment, 125 proteins showed significant reversal in expression compared to the Model group (54 downregulated and 71 upregulated) (**Figure 7C**), suggesting partial correction of disease-associated proteomic dysregulation.

Functional enrichment highlighted key immune−metabolic shifts. GO analysis indicated that downregulated proteins were significantly enriched in terms such as Ruffle, Postsynaptic density, Cellular detoxification of aldehyde, and Response to xenobiotic stimulus (**Figure 7D**). Upregulated proteins were primarily enriched in Cholesterol metabolic process, Complement activation (especially the alternative pathway), Tolerance induction to lipopolysaccharide, Cell surface, and Extracellular space (**Figure 7E**).

KEGG pathway analysis further clarified critical regulatory networks. Downregulated pathways included Notch signaling pathway, Viral protein interaction with cytokine and cytokine receptor, and Ferroptosis (**Figure 7F**). Upregulated pathways were significantly enriched in Cholesterol metabolism, NF-kappa B signaling pathway, Complement and coagulation cascades, and Glycosphingolipid biosynthesis - globo and isoglobo series (**Figure 7G**).

To further explore functional associations among differentially expressed proteins, protein-protein interaction (PPI) networks were constructed. In the Model-vs-Control network (**Figure 7H**), differentially expressed proteins were significantly enriched in modules related to inflammatory response, apoptosis regulation, and oxidative stress. In contrast, the Wilforine-vs-Model network (**Figure 7I**) showed a shift in core hub proteins, with significant enrichment in complement system regulation, lipid metabolism and transport, and cell surface receptor signaling. Comparison of the two networks visually demonstrated that wilforine intervention shifted the core of the PPI network from pro-inflammatory and pro-apoptotic modules represented by proteins such as Casp3, Casp8, and Nfkbia, toward immunomodulatory and lipid homeostasis modules centered on Cfb, Apoa1, and Apoa4. This shift corroborates, at a systems level, the molecular mechanism by which wilforine reverses the disease state by modulating specific functional modules.

In summary, integrated proteomics systematically demonstrates that Wilforine orchestrates a dual immunometabolic reprogramming: it enhances cholesterol and glycosphingolipid biosynthesis to remodel the composition and function of membrane lipid rafts—key platforms for immune signaling—while concurrently fine−tuning major inflammatory/immune networks such as NF−κB and the complement system. These changes collectively inhibit the formation of lipid−raft−dependent resorptive structures (e.g., ruffles) and provide a comprehensive molecular rationale for Wilforine’s alleviation of inflammatory bone marrow edema and destruction.

### 3.7 Transcriptomic profiling demonstrates wilforine’s reprogramming of osteoclast differentiation and cytokine-driven immune signaling

**Figure 8:**
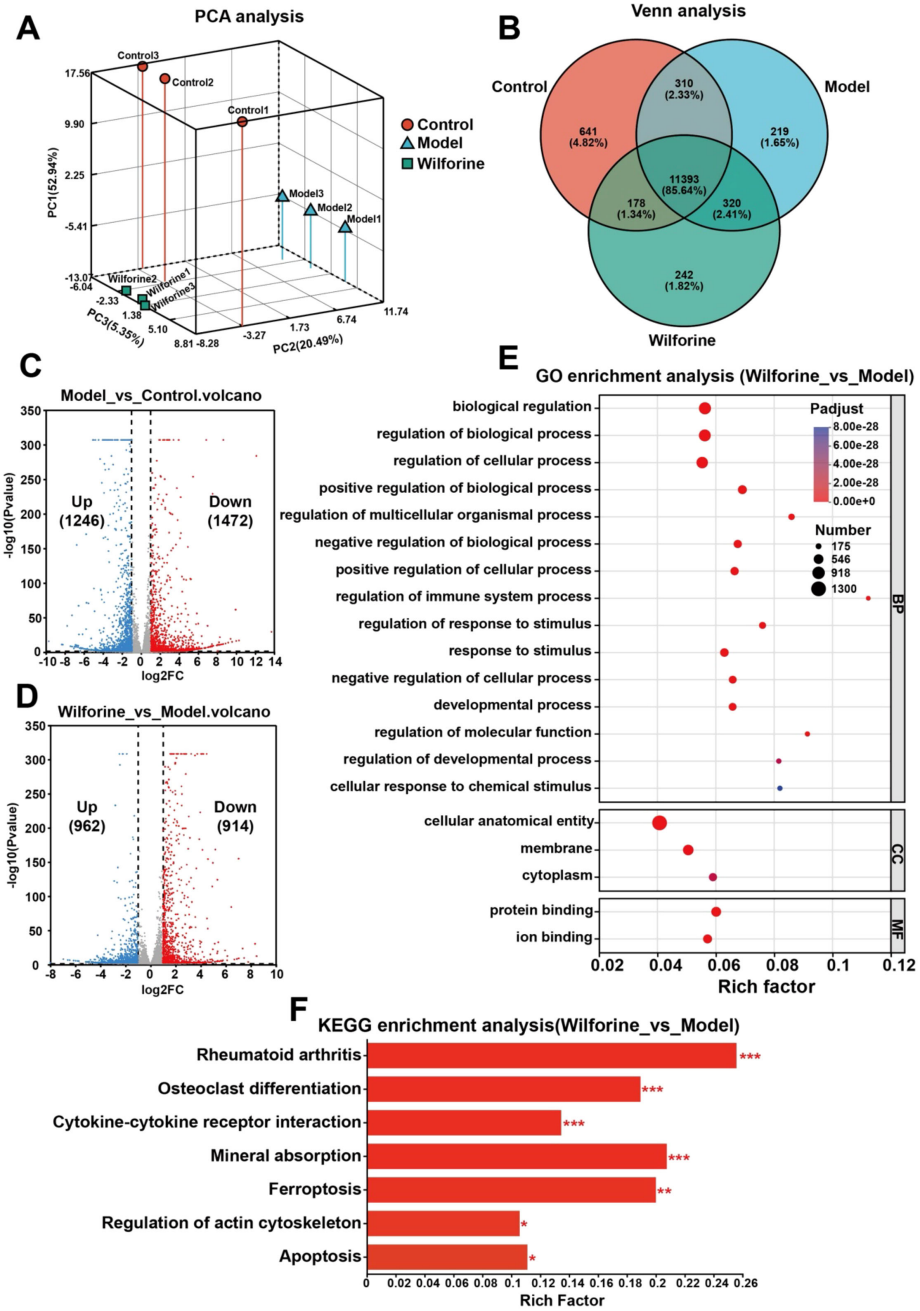
RNA-Seq sequencing reveals the mechanism by which Wilforine regulates osteoclast differentiation. **(A)** Principal component analysis (PCA) of the Control, Model, and Wilforine-treated groups; **(B)** Venn diagram analysis of the Control, Model, and Wilforine-treated groups; **(C)** Volcano plot of differentially expressed genes between the Model and Control groups; **(D)** Volcano plot of differentially expressed genes between the Wilforine-treated and Model groups; **(E)** GO functional enrichment analysis of differentially expressed genes between the Wilforine-treated and Model groups; **(F)** KEGG pathway enrichment analysis of differentially expressed genes between the Wilforine-treated and Model groups.

To elucidate the immunomodulatory mechanism by which wilforine regulates osteoclast differentiation at the transcriptional level, we performed RNA-seq transcriptomic analysis. Principal component analysis (PCA) showed that samples from the Control, Model, and Wilforine groups were distinctly separated into three independent clusters in two-dimensional space (**Figure 8A**), indicating unique gene expression profiles for each group. Venn diagram analysis further revealed a substantial number of uniquely expressed genes among the three groups, with 641, 219, and 242 unique genes identified in the Control, Model, and Wilforine groups, respectively (**Figure 8B**). Differential gene expression analysis indicated that compared to the Control group, the Model group exhibited significant changes in 2718 genes (1246 up-regulated and 1472 down-regulated) (**Figure 8C**). In contrast, wilforine intervention reversed the expression of 1876 genes compared to the Model group (962 up-regulated and 914 down-regulated) (**Figure 8D**), demonstrating its broad transcriptional reprogramming capacity in an inflammatory disease context.

Functional enrichment analysis of the differentially expressed genes (DEGs) between the Wilforine and Model groups was conducted to reveal the core regulatory network. GO analysis showed that the DEGs were significantly enriched in broad biological regulation processes, including Biological regulation, Positive/Negative regulation of biological process, Regulation of cellular process, Regulation of immune system process, and Response to stimulus. Regarding cellular components, they were mainly enriched in the Membrane and Cytoplasm. In terms of molecular function, they were concentrated in Protein binding and Ion binding (**Figure 8E**). These results suggest that wilforine intervention involves multi-level and multifaceted reprogramming of cellular functions.

Key KEGG pathway enrichment analysis revealed that the DEGs were significantly enriched in multiple signaling pathways closely related to disease pathology and treatment (**Figure 8F**), primarily including: Rheumatoid arthritis, Osteoclast differentiation, Cytokine-cytokine receptor interaction, Ferroptosis, and Regulation of actin cytoskeleton.

In summary, transcriptomic profiling confirms that Wilforine extensively reverses disease−associated gene dysregulation, with a core focus on suppressing osteoclast differentiation and modulating cytokine−mediated immune crosstalk. This provides a strong transcriptional foundation for further mechanistic investigation into how Wilforine, potentially via key immunoregulatory pathways such as JAK−STAT, remodels the lipid raft−based immune microenvironment and membrane protein function to inhibit osteoclast activation.

### 3.8 Wilforine disrupts lipid raft integrity and inhibits osteoclast fusion by suppressing the JAK−STAT−stomatin immunoregulatory axis

**Figure 9:**
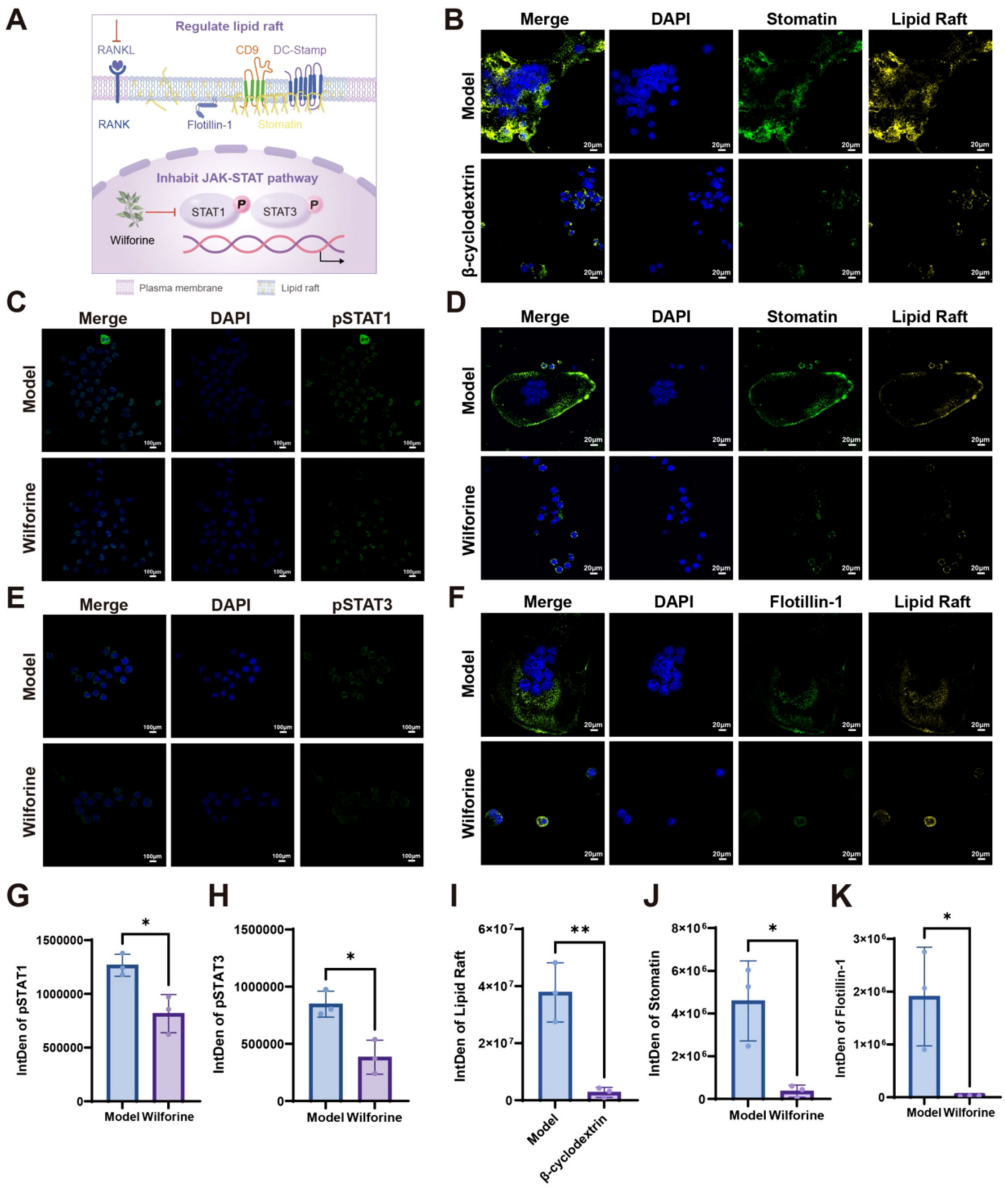
Wilforine inhibits osteoclast fusion by downregulating lipid raft−associated proteins. **(A)** Schematic diagram of the JAK−STAT signaling mechanism; **(B)** β−Cyclodextrin (β−CD) disrupts CT-B–labeled lipid raft distribution and reduces Stomatin signal intensity in osteoclasts; **(C)** Wilforine downregulates pSTAT1 protein expression; **(D)** Wilforine inhibits the expression of the lipid raft−associated protein Stomatin and reduces lipid raft labeling in osteoclasts; **(E)** Wilforine downregulates pSTAT3 protein expression; **(F)** Wilforine inhibits the expression of the lipid raft−associated protein Flotillin−1 and reduces lipid raft labeling in osteoclasts; **(G)** Quantitative analysis of pSTAT1 downregulation by Wilforine; **(H)** Quantitative analysis of pSTAT3 downregulation by Wilforine; **(I)** Quantitative analysis lipid raft labeling inhibition by β−CD; **(J)** Quantitative analysis of lipid raft labeling inhibition by Wilforine in osteoclasts; **(K)** Quantitative analysis of Flotillin−1 suppression by Wilforine in osteoclasts. (mean ± SD, n = 3, ^*^P < 0.05, ^**^P < 0.01).

To elucidate the mechanism by which Wilforine modulates the osteoclast membrane lipid raft microenvironment—a key platform for immunoreceptor signaling—we investigated its impact on the upstream JAK−STAT pathway, a central mediator of inflammatory and immune responses, and the downstream lipid raft scaffold protein Stomatin, as summarized in **Figure 9A**. First, to validate the essential role of intact lipid rafts in osteoclast fusion, β-cyclodextrin (β-CD) was used to deplete membrane cholesterol. Treatment with 1 mM β−CD for 12 h effectively disrupted lipid raft microdomains, evidenced by a marked reduction in CT−B-labeled punctate structures (**Figures 9B, I**), and concurrently decreased osteoclast multinucleation, validating lipid rafts as a structurally required element for the fusion process in inflammatory osteoclastogenesis.

To assess Wilforine’s effect, osteoclasts were treated with 1 mM Wilforine for 12 h. Wilforine treatment led to a significant reduction in the fluorescence intensity and aggregated distribution of CT−B-labeled lipid rafts on the cell membrane. Furthermore, immunofluorescence staining revealed that Wilforine markedly reduced the membrane expression and clustered patterning of the key lipid raft-associated proteins Stomatin and Flotillin-1 (**Figures 9D, F, J, K**). These results indicate that Wilforine significantly alters the composition and structural integrity of osteoclast membrane lipid rafts, targeting the expression and localization of their core scaffold proteins.

To link these effects to upstream signaling, we examined the JAK-STAT pathway. Immunofluorescence analysis showed that Wilforine treatment significantly reduced the expression of phosphorylated STAT1 and STAT3 (pSTAT1, pSTAT3) (**Figures 9C, E, G, H**). This inhibition of the JAK−STAT cascade, coupled with the downregulation of its downstream effector Stomatin, supports a coherent mechanism: Wilforine attenuates JAK−STAT−driven immunoinflammatory signaling, leading to reduced expression of the raft−stabilizing protein Stomatin, consequent destabilization of lipid raft microdomains, and ultimately impairment of the fusion machinery essential for osteoclast multinucleation. Collectively, these findings uncover a potential immunoregulatory axis—JAK−STAT−Stomatin−lipid raft—that appears to mediate the membrane−targeted, anti−osteoclastic action of Wilforine.

## 4 Discussion

The excessive activation and fusion of osteoclasts are central pathological drivers of inflammatory bone destruction in immune-mediated osteopathies such as SAPHO syndrome. This study delineates a novel, immunomodulatory mechanism by which the natural compound Wilforine alleviates bone erosion. Our integrated findings demonstrate that Wilforine reprograms immunometabolic pathways and disrupts the “JAK−STAT – Stomatin – Lipid Raft” immunoregulatory axis, thereby inhibiting osteoclast fusion and bone-resorptive activity.

Our *in vivo* results provide compelling evidence that Wilforine significantly alleviates bone erosion, marrow edema, and immune cell-driven synovitis in the Pstpip2cmo mouse model. The reduction in arthritis scores, paw thickness, and histopathological damage, coupled with the downregulation of key pro-inflammatory cytokines (IL−1β, IL−6) and upregulation of the anti-inflammatory mediator IL−10, establishes Wilforine’s dual immunomodulatory and bone-protective functions. This systemic immunomodulatory property is consistently observed across inflammatory models (52, 53). For instance, Huang et al. demonstrated in a collagen-induced arthritis (CIA) model that Wilforine reduced arthritis severity and significantly lowered serum levels of IL−6, IL−1β, and TNF−α (19), highlighting its reproducible suppression of core inflammatory drivers in immune−mediated bone disease. Furthermore, network pharmacology analysis suggests Wilforine may target signaling nodes such as EGFR and SRC, which are implicated in immune cell activation and osteoclastogenesis, potentially contributing to its broad-spectrum immunoregulatory efficacy. Relevant studies also indicate that active components of Tripterygium wilfordii (including Wilforine) can inhibit functional proteins such as Organic Anion Transporting Polypeptide 1B1 (OATP1B1) (54), providing further support for its capacity for direct modulation of membrane-associated transport and signaling proteins involved in immune cell function. Collectively, this evidence positions the multi-pathway immunomodulatory action of Wilforine as the critical mechanistic foundation for its bone-protective effects.

A key finding of this study is the identification of cell membrane lipid rafts as a critical immunomodulatory microenvironmental target of Wilforine. Lipid rafts are essential platforms for clustering immune receptors and signaling molecules and facilitating cell-cell fusion (55). We found that Wilforine, similar to the lipid raft disruptor β-cyclodextrin, potently inhibited osteoclast multinucleation *in vitro*. This effect was functionally linked to the impaired raft-dependent localization and function of key fusogenic proteins, CD9 and DC-STAMP, within the inflammatory osteoclastogenesis context. The established role of CD9 in lipid rafts for osteoclast fusion (29) aligns with our observation of its disrupted function upon Wilforine treatment. The downregulation of critical lipid raft scaffold proteins, Stomatin and Flotillin-1, both *in vivo* and *in vitro*, provides a mechanistic explanation for the disruption of these immune-signaling membrane microdomains. Importantly, Stomatin has been directly shown to enhance cell fusion, where its depletion inhibits osteoclast fusion without affecting differentiation marker expression (56). Our finding that Wilforine downregulates Stomatin offers a specific molecular link to its anti-fusion effect via modulation of an immune-related signaling scaffold. Furthermore, the integrity of lipid rafts is known to be crucial not only for fusion but also for the activity of osteoclast resorptive machinery, such as V-ATPase (38), suggesting that Wilforine’s raft-disrupting action may have broad inhibitory consequences on osteoclast function in inflammatory bone resorption. This positions pharmacological modulation of lipid raft assembly as a viable and innovative immunomodulatory strategy to curb pathological osteoclast fusion.

The strength of this study lies in the systematic integration of proteomics and transcriptomics, which revealed that Wilforine induces a broad reversal of disease-associated dysregulation through immunometabolic reprogramming. The omics data demonstrate that Wilforine significantly upregulates pathways related to cholesterol metabolism and glycosphingolipid biosynthesis. Given that cholesterol and sphingolipids are fundamental structural components of lipid rafts—defining their biophysical properties and organizing membrane-resident signaling molecules (57), their metabolic rewiring directly impacts raft composition, stability, and immune-signaling capacity. This aligns with the therapeutic concept of modulating lipid raft assembly by targeting cholesterol and sphingolipid metabolism (58). Concurrently, Wilforine finely tuned pivotal immune-inflammatory networks, including the NF-κB pathway and the complement system, both of which are known to be spatially organized within lipid raft platforms. Lipid rafts serve as critical scaffolds for the assembly of inflammatory signaling complexes, such as those mediating IL-6 signaling (59), and are directly involved in key immunoregulatory pathways like NF-κB activation (60). This systemic remodeling of the “lipid-immune interface” underscores the pleiotropic immunomodulatory nature of Wilforine. It explains its profound impact on osteoclast biology by simultaneously destabilizing the membrane microdomains essential for cell fusion and attenuating the raft-coordinated inflammatory signals that drive osteoclastogenesis.

We further elucidated the upstream signaling event linking Wilforine to lipid raft disruption. Our data indicate that Wilforine inhibits the activation (phosphorylation) of STAT1 and STAT3. The JAK−STAT pathway is a master regulator of cytokine signaling and immune responses (61–63). Clinically approved JAK inhibitors exert their therapeutic effects primarily through direct inhibition of JAK kinase activity, thereby suppressing downstream STAT phosphorylation and inflammatory transcriptional programs (64–67). In our study, Wilforine treatment was associated with reduced pSTAT1 and pSTAT3 signal intensity in osteoclasts. However, unlike classical JAK inhibitors that directly target kinase activity (68), our findings suggest that Wilforine may influence JAK-STAT signaling in parallel with modulation of membrane microdomain organization, including alterations in lipid raft labeling and lipid raft–associated scaffold proteins. This distinction highlights a potential difference in regulatory level: while JAK inhibitors act directly on kinase activity, Wilforine may operate at the membrane microenvironment level, which in turn could affect cytokine receptor organization and signal initiation. Beyond direct kinase inhibition, the efficient initiation of JAK-STAT signaling is highly dependent on the integrity of lipid raft microdomains, which serve as platforms for cytokine receptor assembly, such as the IL-2 receptor and IL-7 receptor, and for the subsequent recruitment and activation of JAK kinases (69, 70). Critically, perturbations in lipid raft composition or structure can directly impair immune signal transduction via the JAK−STAT axis. For instance, arachidonic acid accumulation in lipid rafts displaces signaling components and inhibits JAK−STAT phosphorylation (71), and 27-hydroxycholesterol disrupts lipid rafts to block IL-6-JAK-STAT3 signaling (72). This establishes a precedent where altering the lipid raft microenvironment leads to the suppression of this pathway. Conversely, activation of the JAK-STAT pathway itself can promote lipid raft-dependent processes, such as the increased cell surface localization of proteins like ecto-adenosine deaminase via mechanisms involving lipid raft-mediated exocytosis (73).

Crucially, we identified the immunomodulatory lipid raft scaffold protein Stomatin as a functional downstream target of this pathway. Wilforine−induced suppression of JAK−STAT−mediated immune signaling led to the downregulation of Stomatin expression. As a key scaffold, Stomatin is vital for maintaining lipid raft architecture—a membrane platform essential for immune receptor clustering—and for recruiting fusion machinery in osteoclasts. Therefore, the “JAK−STAT – Stomatin – Lipid Raft” axis represents a coherent immunoregulatory cascade through which Wilforine exerts its anti−fusion effects. This axis illustrates a bidirectional immune−membrane crosstalk: lipid rafts facilitate JAK−STAT−dependent cytokine signaling (74, 75), and activated JAK−STAT signaling, in turn, can promote the expression or functional deployment of lipid raft−organizing proteins such as Stomatin. By inhibiting the upstream JAK−STAT immune trigger, Wilforine disrupts this pro−inflammatory positive feedback loop, leading to the collapse of the lipid raft microdomains that are indispensable for osteoclast fusion in inflammatory bone disease.

Current therapies for inflammatory bone diseases, such as bisphosphonates and biologics, often face limitations regarding side effects, cost, and variable immunomodulatory efficacy. This study positions Wilforine as a promising immunomodulatory, multi−targeted agent with a novel mechanism of action. Unlike direct inhibitors of osteoclast enzymes or cytokines, Wilforine targets the cellular membrane microenvironment—specifically lipid rafts, which are critical platforms for immune signal integration during osteoclastogenesis.

## 5 Summary and outlook

This study systematically elucidates the bone-protective effects and immunomodulatory mechanisms of the natural compound Wilforine in an inflammatory osteopathy model by integrating *in vivo* experiments, multi-omics analyses, and *in vitro* functional validation. The results demonstrate that Wilforine significantly alleviates bone erosion, marrow edema, and joint inflammation in Pstpip2^cmo^ mice. This therapeutic effect is associated with the inhibition of pro-inflammatory cytokines (e.g., IL-1β, IL-6) and the promotion of the anti-inflammatory mediator IL-10. Mechanistically, Wilforine suppresses the JAK-STAT signaling pathway, leading to the downregulation of the key lipid raft scaffold protein Stomatin and the consequent disruption of membrane lipid raft integrity. As critical platforms for immune signal transduction, the destabilization of lipid rafts impairs the localization and function of essential fusogenic proteins, CD9 and DC-STAMP, ultimately inhibiting osteoclast multinucleation and bone-resorptive activity. Multi-omics profiling further reveals that Wilforine induces systemic reprogramming of cholesterol and sphingolipid metabolism while modulating pivotal immune-inflammatory networks, including the NF-κB pathway and the complement system. Consequently, this work not only defines a novel “JAK-STAT–Stomatin–lipid raft” immunoregulatory axis but also establishes lipid rafts as a potential therapeutic microenvironmental target, providing a solid theoretical and experimental foundation for the clinical translation of Wilforine.

Certainly, this study has some limitations. First, while the coordinated suppression of JAK-STAT activation, reduction of Stomatin expression, and disruption of lipid raft organization collectively support the proposed regulatory axis, the present evidence is primarily based on correlative expression and imaging analyses. Definitive causal hierarchy will require future genetic and functional validation, including rescue experiments and biochemical assessment of upstream JAK activity. Second, the Pstpip2cmo mouse model, a spontaneous mutation with Mendelian recessive inheritance, presents inherent breeding difficulties that resulted in relatively small group sizes (n=3–4), which consequently precluded a statistically valid sex-stratified analysis. Third, the *in vitro* experiments were conducted using only a single concentration of Wilforine; therefore, a comprehensive dose-response analysis remains to be performed. Furthermore, the apparent paradox between the upregulation of cholesterol/glycosphingolipid metabolic pathways and the observed reduction in lipid raft integrity remains to be definitively resolved, as direct biochemical evidence, such as membrane cholesterol quantification and lipid raft fractionation, was not performed in this study. Finally, the initial molecular target of Wilforine’s direct action (which may be a membrane receptor or a specific kinase) has not yet been fully identified; its safety and efficacy in humans require further clinical validation.

Future research should therefore prioritize: 1) the identification of direct molecular targets of Wilforine using chemical proteomics–based affinity probe enrichment coupled with mass spectrometry screening, followed by orthogonal target engagement validation and functional confirmation; 2) validation of its efficacy in additional bone erosion models such as collagen-induced arthritis; 3) exploration of its combinational therapeutic potential with established anti-resorptive agents; and 4) conducting larger, adequately powered studies to enable sex-stratified analyses and to confirm the reproducibility of these findings. In addition, pharmacologically oriented validation, including dose–response assessment across lower concentration ranges and cytotoxicity profiling, will be essential to refine exposure relevance and translational interpretation. Despite these limitations, the present findings establish a mechanistic framework for understanding the immunomodulatory effects of Wilforine and provide an important theoretical basis for developing novel therapeutic strategies targeting the lipid raft microenvironment in bone metabolic diseases.

## Data Availability

The datasets presented in this study can be found in online repositories. The names of the repository/repositories and accession number(s) can be found below: PXD073543 (iProX) and PRJNA1419546 (Bioproject, NCBI).

## Glossary

JAK-STAT: Janus kinase–signal transducer and activator of transcription
SAPHO: synovitis, acne, pustulosis, hyperostosis, osteitis
TRAP: tartrate-resistant acid phosphatase
Micro-CT: micro-computed tomography
HE: hematoxylin and eosin
β-CD: β-cyclodextrin
DEGs: differentially expressed genes
PPI: protein-protein interaction
GO: Gene Ontology
KEGG: Kyoto Encyclopedia of Genes and Genomes
EGFR: epidermal growth factor receptor
SRC: proto-oncogene tyrosine-protein kinase SRC
MAPK1: mitogen-activated protein kinase 1
CTSK: cathepsin K
RANKL: receptor activator of nuclear factor kappa-B ligand
DC-STAMP: dendritic cell-specific transmembrane protein
CD9: cluster of differentiation 9
IL-1β: interleukin-1β
IL-6: interleukin-6
IL-10: interleukin-10
STAT: signal transducer and activator of transcription
TNF-α: tumor necrosis factor-alpha
PCA: principal component analysis
RNA-seq: RNA sequencing
LC–MS/MS: liquid chromatography–tandem mass spectrometry
OATP1B1: organic anion transporting polypeptide 1B1
SPF: specific pathogen-free
BSA: bovine serum albumin
PBS: phosphate buffered saline
DAB: 3,3’-diaminobenzidine
HRP: horseradish peroxidase
DAPI: 4’,6-diamidino-2-phenylindole
DMEM: Dulbecco’s Modified Eagle Medium
FBS: fetal bovine serum
DMSO: dimethyl sulfoxide
PEG: polyethylene glycol
SD: standard deviation
ANOVA: analysis of variance
SRA: Sequence Read Archive
