# Supplementary material for: Wilforine attenuates inflammatory osteolysis by suppressing osteoclast fusion through JAK-STAT-stomatin immunoregulatory signaling
Source: Front Immunol. 2026 Mar 13;17:1789493. doi: 10.3389/fimmu.2026.1789493 (PMC13021455; doi:10.3389/fimmu.2026.1789493)
Supplement: Supplementary file 1 [file DataSheet1.docx]

**Supporting Information**

**Wilforine Attenuates Inflammatory Osteolysis by Suppressing Osteoclast Fusion through JAK-STAT-Stomatin Immunoregulatory Signaling**

*Shaohui Geng^1,†^, Yiwei Guan^2,†^,* *Zi Ye^2,†^, Dongdong Zhao^1,†,*^, Yijin Jiang^2^,* *Jingyuan Fu^2^,* *Han Sheng^3^, Shuhan Yang^4^, Hongxu Liu^4^, Fuwen Deng^5^, Shasha Yu^1^, Mureziya Yimingjiang^1^, Yuanhao Wu^6,*^, Chen Li^7,*^, Guangrui Huang^1,*^*

^1^ School of Life Science, Beijing University of Chinese Medicine, Beijing 100029, China;

^2^ School of Chinese Materia Medica, Beijing University of Chinese Medicine, Beijing, China, Beijing 100029, China;

^3^ Dongzhimen Hospital, Beijing University of Chinese Medicine, Beijing, China;

^4^ School of Traditional Chinese Medicine, Beijing University of Chinese Medicine, Beijing 100029, China;

^5^ School of Nursing, Beijing University of Chinese Medicine, Beijing 100029, China;

^6^ First Teaching Hospital of Tianjin University of Traditional Chinese Medicine，Tianjin, 300381, China;

^7^ Shanghai GuangHua Hospital of Integrated Traditional Chinese and Western Medicine, Shanghai University of Traditional Chinese Medicine, 200052, China.

^†^These authors have contributed equally to this work and share first authorship.

**^*^Corresponding Authors:**

Guangrui Huang: [hgr@bucm.edu.cn](mailto:hgr@bucm.edu.cn), 010-53912159, School of Life Science, Beijing University of Chinese Medicine, Beijing 100029, China;

Chen Li: [casio1981@163.com](mailto:casio1981@163.com), 13810988688, Shanghai GuangHua Hospital of Integrated Traditional Chinese and Western Medicine, Shanghai University of Traditional Chinese Medicine, 200052, China;

Yuanhao Wu: [doctor.wuyh@gmail.com](mailto:doctor.wuyh@gmail.com), First Teaching Hospital of Tianjin University of Traditional Chinese Medicine，Tianjin, 300381, China;

Dongdong Zhao: zhaodd@bucm.edu.cn, School of Life Science, Beijing University of Chinese Medicine, Beijing 100029, China.


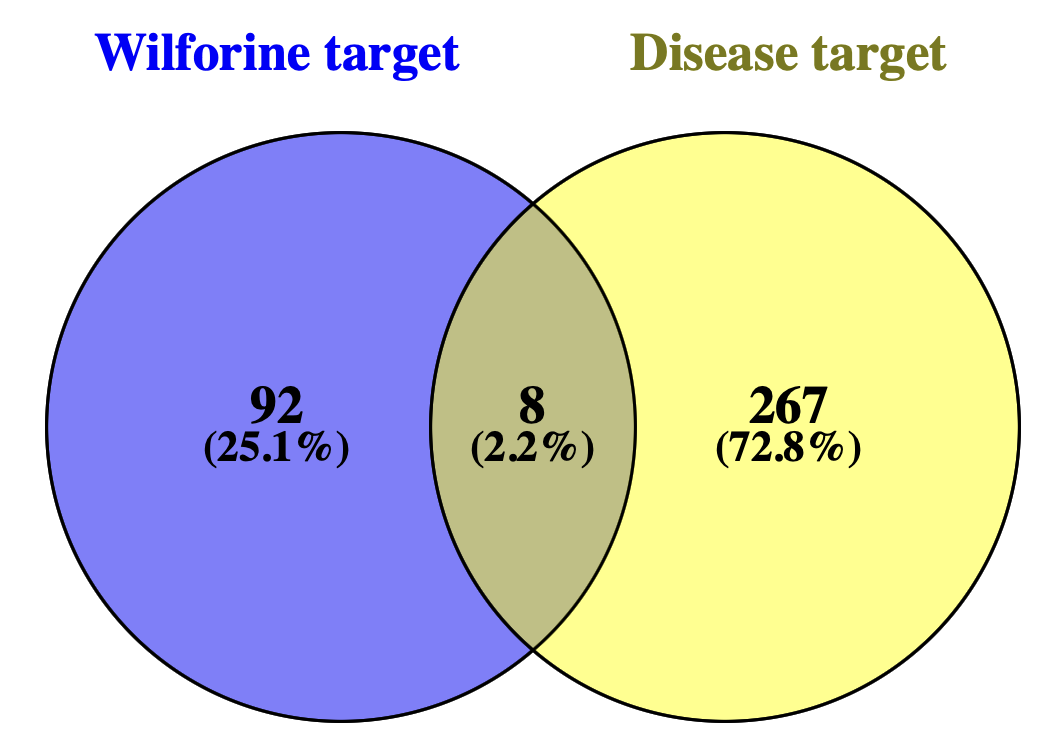


**Sup Figure 1.** Venn diagram illustrating the intersection between Wilforine-related targets and disease-related targets.


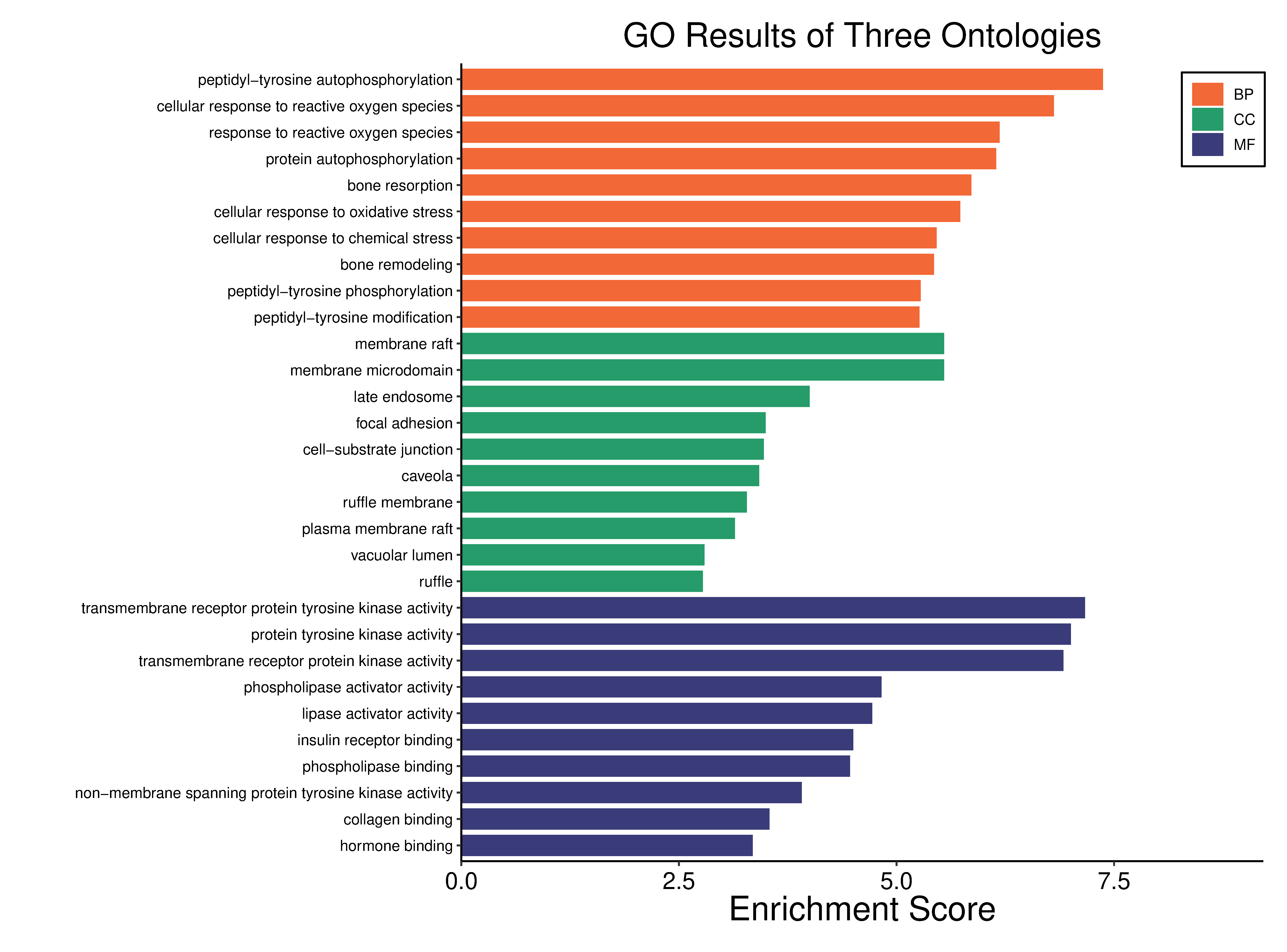


**Sup Figure 2.** GO functional enrichment analysis results of the overlapping targets.


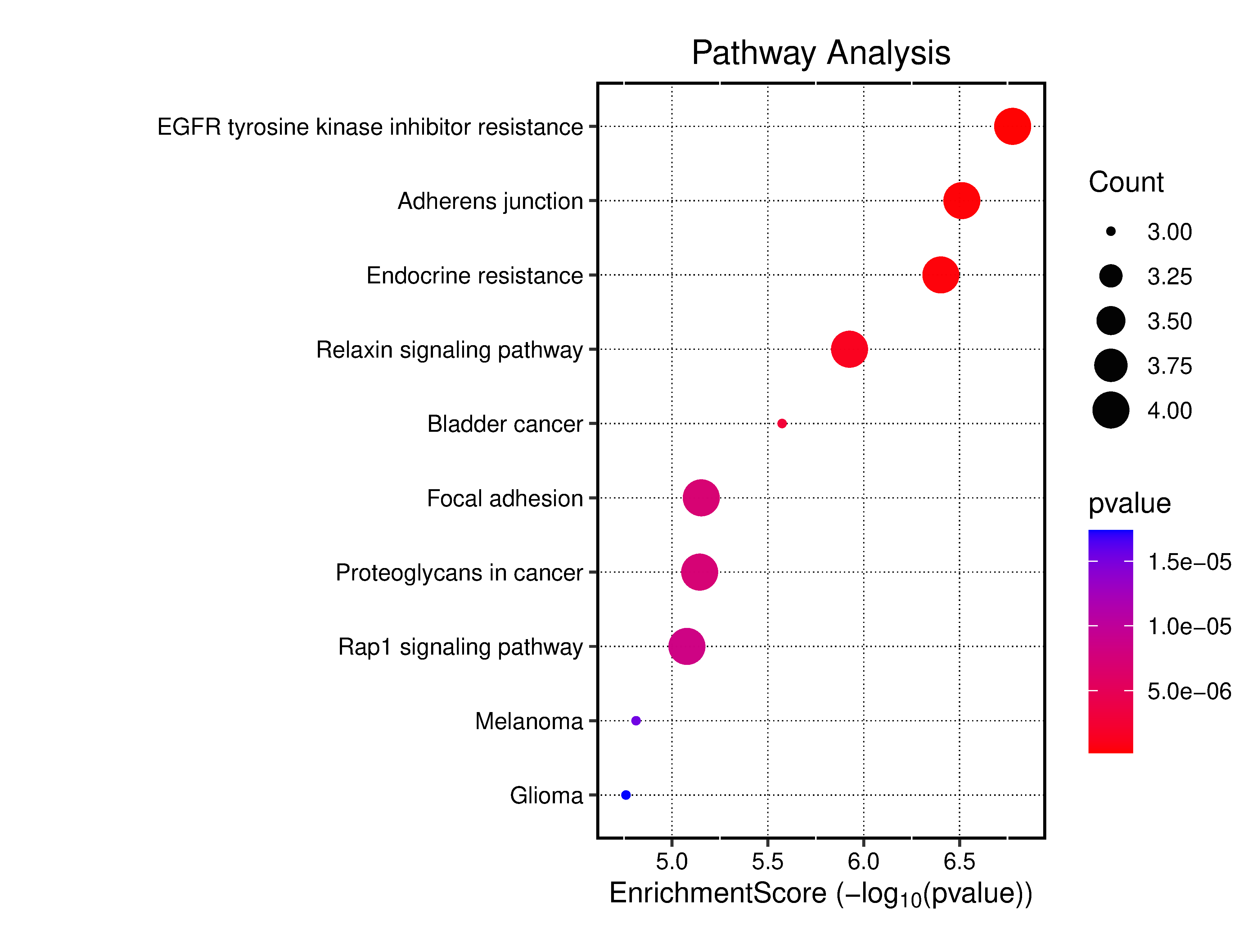


**Sup Figure 3.** Bubble plot of KEGG pathway enrichment analysis for the overlapping targets (bubble size indicates the number of enriched targets; color represents statistical significance).

**
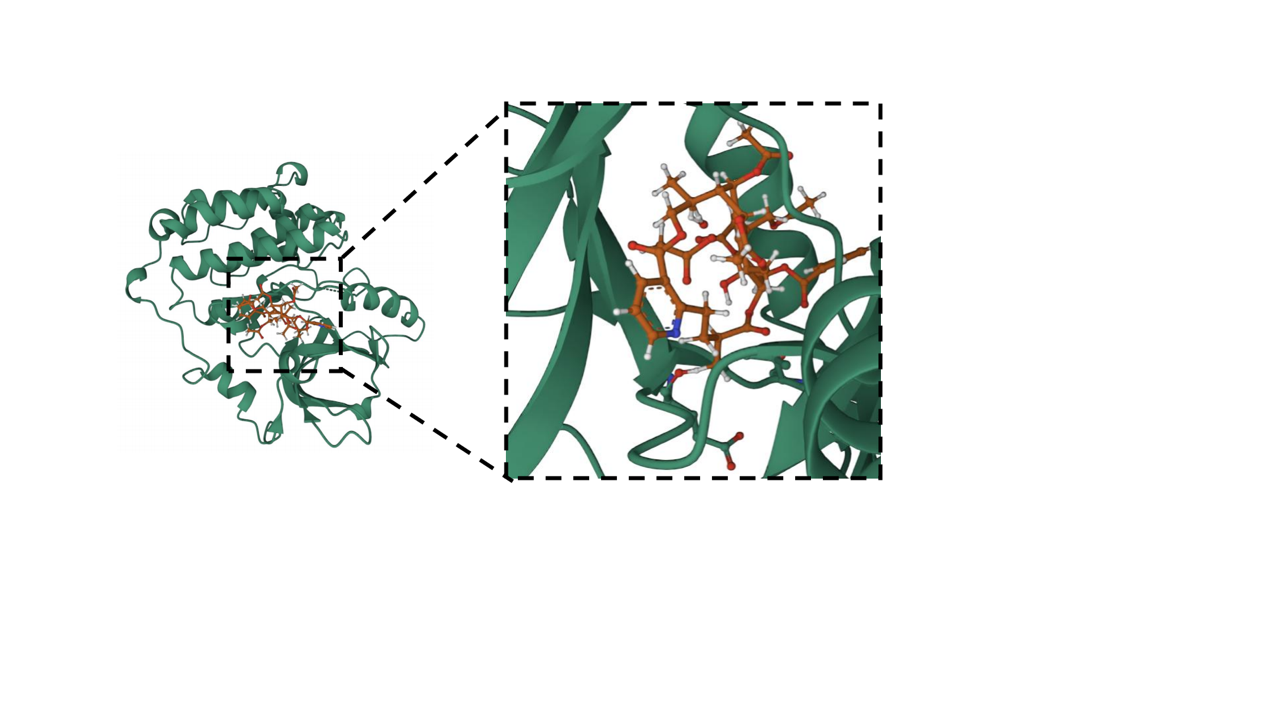
**

**Sup Figure 4.** Molecular docking results of wilforine with EGFR (PDB ID: 6C9D), showing a predicted binding energy of −7.952 kcal/mol.

**
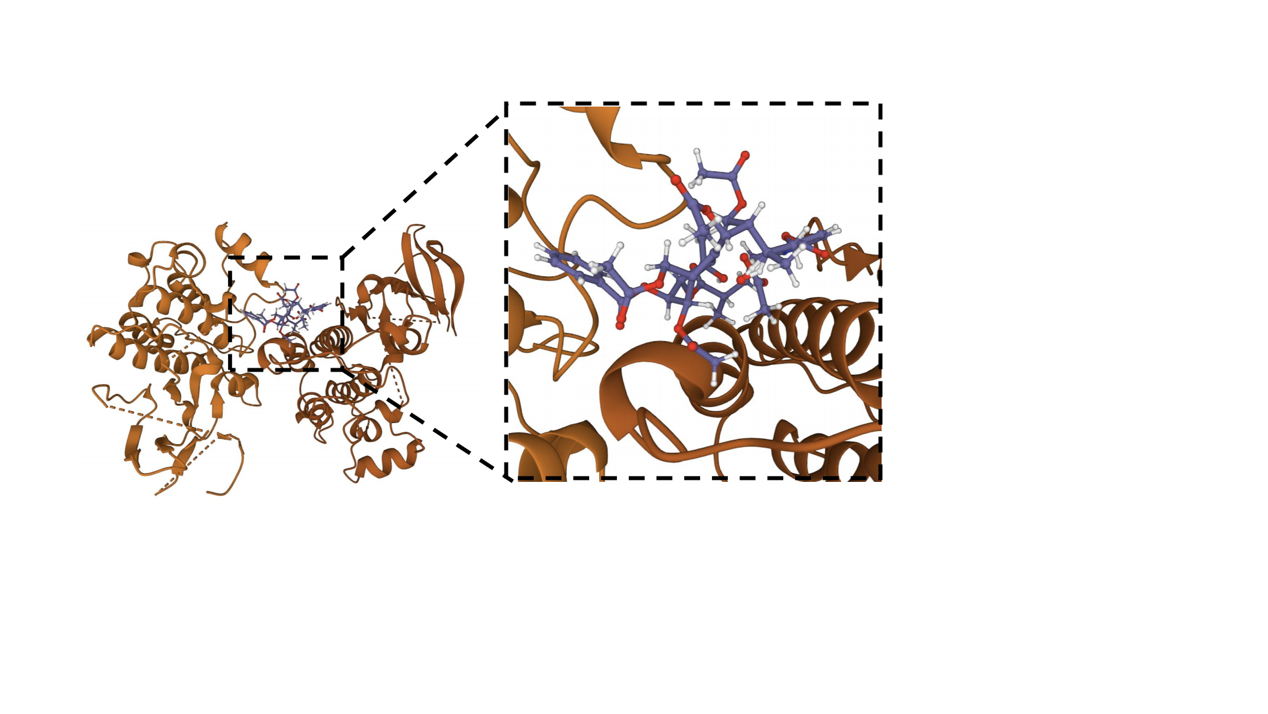
**

**Sup Figure 5.** Molecular docking results of wilforine with SRC (PDB ID: 1YOJ), with a predicted binding energy of −7.631 kcal/mol.


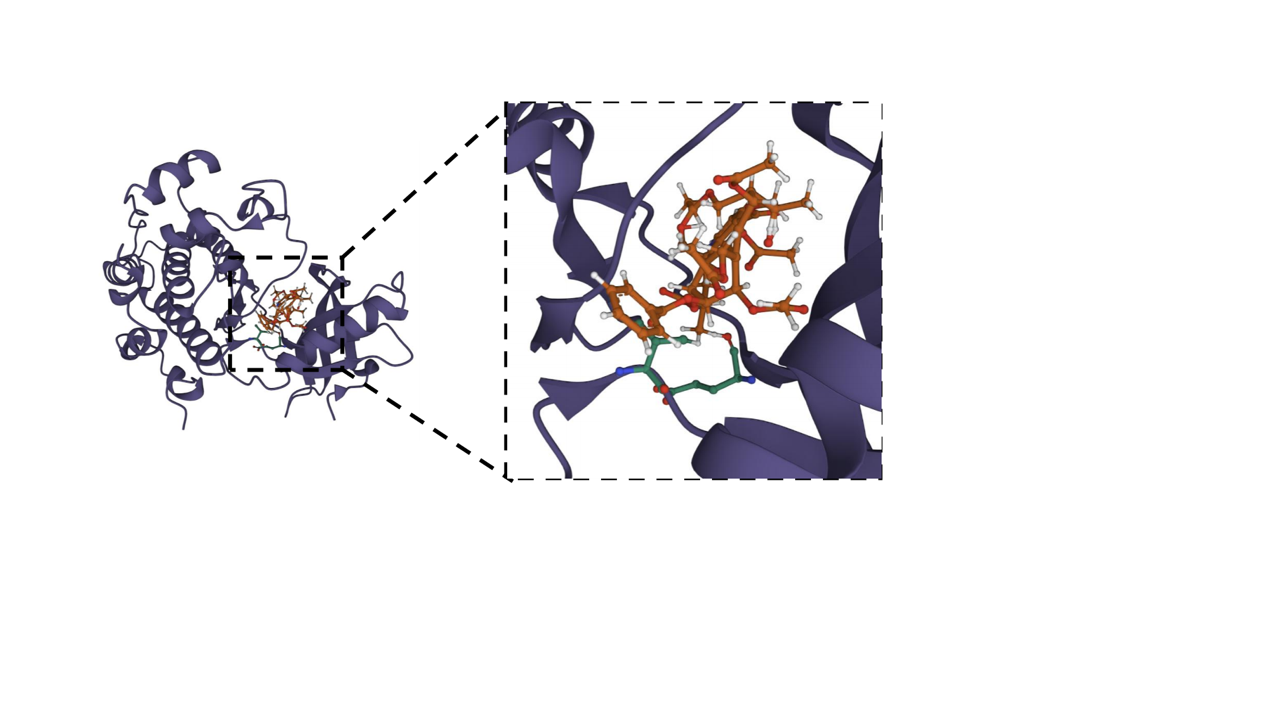


**Sup Figure 6.** Molecular docking results of wilforine with CTSK (PDB ID: 3W2S), with a predicted binding energy of −7.473 kcal/mol.
